# Phage-ICE tandems facilitate bacterial adaptation and antimicrobial resistance spread

**DOI:** 10.1126/sciadv.aed3416

**Published:** 2026-10-09

**Authors:** Jinhu Huang, Jiaqi Zhao, Pengchao Zhan, Xingyang Dai, Xinming Pan, Zhenmiao Li, Wenbin Zhao, Xiaoli Shi, Xiaoming Wang, Shengyong Mao, Jiale Ma, Liping Wang, Jinxin Liu

**Affiliations:** ^1^ Sanya Institute of Nanjing Agricultural University, Sanya 572025, China.; ^2^ MOE Joint International Research Laboratory of Animal Health and Food Safety, Risk Assessment Center of Veterinary Drug Residue and Antimicrobial Resistance, Center for Veterinary Drug Research and Evaluation, College of Veterinary Medicine, Nanjing Agricultural University, Nanjing 210095, China.; ^3^ Laboratory of Gastrointestinal Microbiology, Jiangsu Key Laboratory of Gastrointestinal Nutrition and Animal Health, National Center for International Research on Animal Gut Nutrition, College of Animal Science and Technology, Nanjing Agricultural University, Nanjing 210095, China.; ^4^ WOAH Reference Lab for Swine Streptococcosis, Nanjing 210095, China.; ^5^ The Air Force Hospital of Eastern Theater of PLA, Nanjing 210002, China.; ^6^ National Experimental Teaching Demonstration Center for Animal Science, Nanjing 210095, China.

## Abstract

Phages and integrative and conjugative elements (ICEs) drive bacterial evolution, but how their interplay shapes bacterial adaptation and antimicrobial resistance (AMR) dissemination remains unclear. Here, we characterize phage-ICE tandems formed by site-specific accretion of phages and ICEs into cotransferable units. In *Streptococcus*, phage-encoded integrases (IntIE_Phage_) drive stress-inducible excision and circularization, after which ICE machinery mediates conduction of the tandem. When excision is impaired, phage and phage-ICE fragments disseminate by transformation and integrate via RecA-dependent homologous recombination (HR), providing a fail-safe route. This pathway enables cotransfer of flanking chromosomal DNA, generating length-variable integrations that remodel transcriptional programs and increase oxidative stress tolerance. A global screen identified 612 phage-ICE tandems across seven phyla and 135 species, including multidrug-resistant pathogens, with lineage-specific repertoires of AMR, virulence, and metabolic genes. Rather than a hybrid element, these structures represent functional interplay where prophages exploit ICE conjugation while facilitating mobilization of the composite region. By coupling excision, transfer, and recombination, phage-ICEs shape adaptation and accelerate AMR spread.

## INTRODUCTION

Antimicrobial resistance (AMR) threatens effective therapy and public health (*1*, *2*). Central to its propagation is horizontal gene transfer (HGT) mediated by mobile genetic elements (MGEs) such as plasmids, integrative and conjugative elements (ICEs), transposons, integrons, and bacteriophages (*3*–*6*). Traditionally, these MGEs have been associated with distinct routes—conjugation for plasmids and ICEs, transduction for phages, and natural transformation for free DNA. Accumulating evidence indicates, however, that MGEs frequently interact to enable expanded mobility (*7*–*11*). For example, conjugative systems can mobilize nonautonomous elements via shared type IV secretion machinery (*12*, *13*), and phages can use auto- or lateral transduction to circumvent host barriers (*14*–*17*). Moreover, the formation of chimeric infective particles between distinct MGEs has been shown to drastically expand species boundaries for genetic mobilization (*18*). Multifaceted MGEs, such as phage-plasmids, exemplify the functional integration of diverse lifestyles—specifically combining the temperate phage cycle with stable episomal replication (*19*–*23*).

Beyond these interactions, distinct MGEs can integrate at the same chromosomal site, leading to the formation of tandem arrays or composite elements via site-specific accretion (*24*, *25*). Despite this progress, the functional interplay between chromosomally integrated ICEs and prophages within these tandems remains less well defined (*26*). Although both are common in bacterial genomes, their colocalization and assembly into composite structures with expanded transfer modes and cargo retention have been underexplored (*19*, *20*). In *Streptococcus*, a recent work indicates that ICEs can mobilize prophages at appreciable frequencies and that both modules can occupy the same genomic site, forming tandems with interdependent transfer (*27*). Compared with phage-plasmid systems (*19*, *20*, *28*, *29*), the mechanistic basis and generality of this physical coupling are not fully understood. These observations raise two central questions. First, can phage-ICE tandems undergo conduction (cis-mobilization), and what molecular processes govern this mobility and its evolutionary conservation? Second, does this composite configuration simply accelerate AMR dissemination, or does it also confer context-dependent adaptive advantages?

Here, we characterize phage-ICE tandems as cointegrates that combine prophages and ICEs into modular, cotransferable units. Using *Streptococcus* as a model, we show that phage integrases (IntIE_Phage_) facilitate excision not only of prophage modules but also of adjacent ICE and composite phage-ICE tandems. Subsequently, ICE-encoded conjugation mobilizes the excised episomes via conduction (*13*). When IntIE_Phage_-mediated excision is impaired, dissemination proceeds via cotransformation, with phage and phage-ICE fragments integrating through homologous recombination (HR). This HR-coupled route generates strain-specific genomic rearrangements and variable-length donor tracts and is associated with increased oxidative stress tolerance. A survey of ∼320,000 microbial genomes identified 612 phage-ICE tandems across seven phyla and 135 species, including high-risk multidrug-resistant (MDR) pathogens such as *Pseudomonas aeruginosa* and *Staphylococcus aureus*. These data recast MGE cooperativity as a tripartite excision-transfer-recombination system and position phage-ICEs as tandem elements that shape bacterial adaptation and promote the spread of antibiotic resistance.

## RESULTS

### Phage-ICEs expand AMR reservoirs in pathogenic streptococci

Phages and ICEs are classically treated as distinct MGEs mediating transduction and conjugation, respectively. Our analyses reveal their convergence in *Streptococcus* as phage-ICE tandems that act as prominent vehicles for AMR dissemination. These tandems integrate adjacently at conserved recombination hotspots—most notably the *rum* locus—and occur in two orientations (phage-ICE and ICE-phage). This configuration, reminiscent of ICE-ICE tandem arrays identified in *Streptococcus* (*24*, *25*), reflects a process of site-specific accretion. By physically coupling distinct modules, these tandems carry a substantially larger repertoire of AMR and virulence genes than individual elements, thereby increasing the genetic payload and facilitating the efficient codissemination of multiple resistance determinants (Fig. 1 and data S1).

**Fig. 1.**
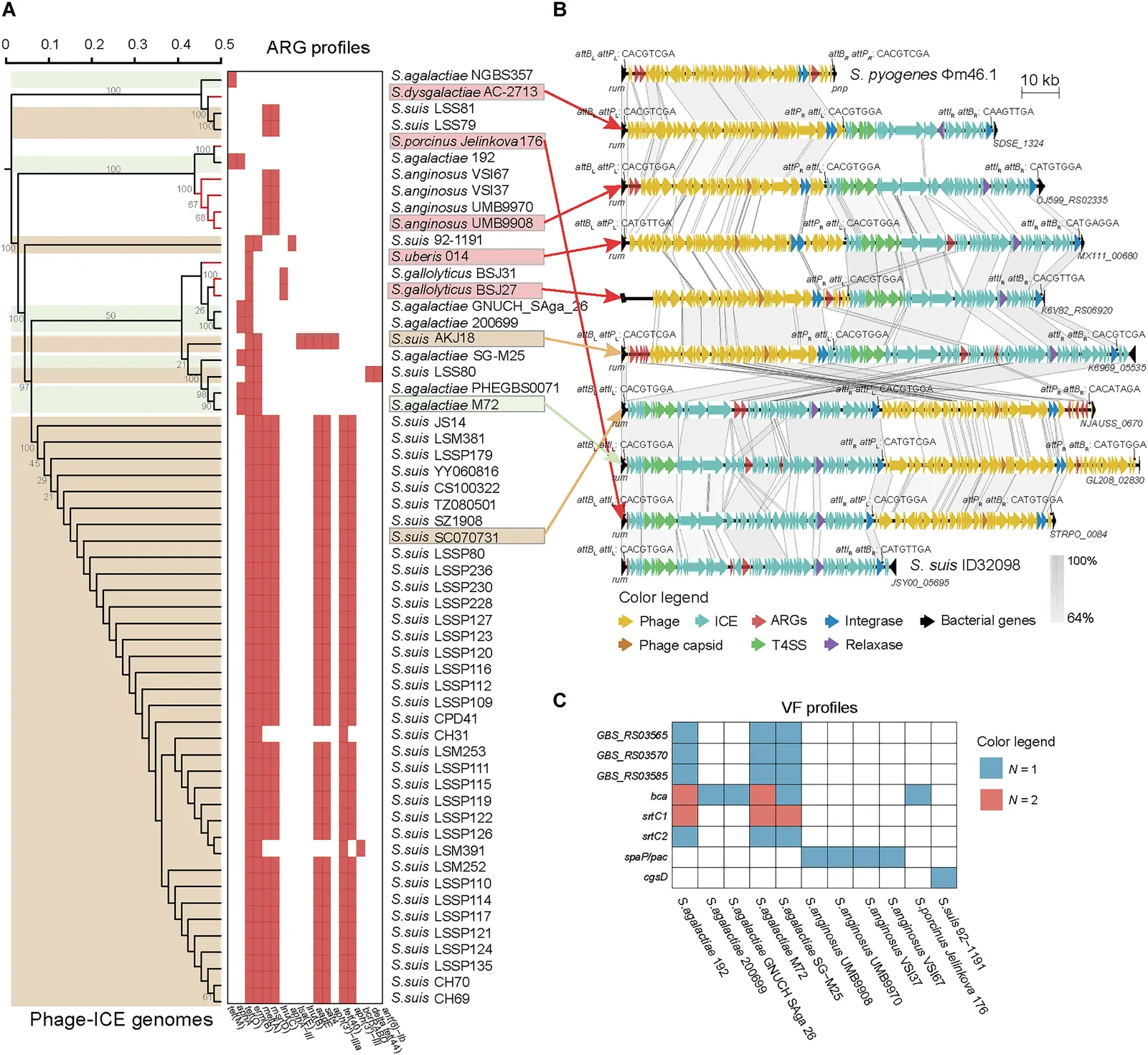
Phylogenetic analysis and genomic organization of phage-ICE tandems. (**A**) (Left) ML phylogeny of 57 phage-ICE sequences with resolved attachment sites. *S. suis* and *S. agalactiae* are shaded light bronze and light mint green, respectively; vermilion leaves indicate other streptococci. Phylogenetic tree with bootstrap values percentages (≥20%) shown at the nodes. (Right) Distribution of ARGs; red indicates presence (detailed in data S1). (**B**) Architectures of representative phage-ICEs across seven *Streptococcus* species. ICE and phage genes are shown as mint green and yellow arrows, respectively, and phage capsid proteins, type IV secretion system (T4SS) components, and relaxases are shown as orange-brown, green, and purple, respectively; ARGs, integrases, and chromosomal genes are shown as red, blue, and black arrows. (**C**) Distribution of virulence factors (VFs). The *x* axis lists VF-positive strains; the *y* axis lists VF categories. Blue denotes one VF; red denotes two VFs.

A systematic screen of GenBank *Streptococcus* genomes (accessed October 2023) identified 114 phage-ICE tandems, 57 of which were fully resolved across attachment sites and boundaries (Fig. 1A, fig. S1, and data S1). Their distribution was skewed toward *S. suis* (64.9%, 37/57), followed by *S. agalactiae* (19.3%) and *S. anginosus* (8.8%). Genetic characterization of the constituent modules revealed that all phage components belonged to the SMphage family—a widespread lineage of streptococcal prophages that had been identified previously (*27*). Meanwhile, the ICE modules displayed high levels of conservation and belonged to the Tn*5252*/Tn*1549* family, which carries relaxases belonging to the MOBP1 family (data S1). Despite broadly conserved architectures (Fig. 1B and fig. S2A), phylogenies of phage modules (*orf13-55* and *intIE*_Phage_), and ICE core genes (*virB4*, *virD4*, *relaxase*, and *int*_ICE_) show partially discordant topological patterns, suggesting distinct evolutionary trajectories and frequent modular recombination (Fig. 1A and fig. S2, B and C).

Phage submodules (47.9 to 88.6 kb) and ICE submodules (40.0 to 76.0 kb) carried a complementary cargo. The phage submodules, all belonging to the SMphage family, predominantly (94.7%) encoded MDR genotypes—most frequently the *mef*(A)+*msr*(D)+*aph*(3′)-III+*sat4*+*aadE*+*erm*(B) (*N* = 34)—conferring resistance to macrolides, aminoglycosides, and streptogramins. These AMR determinants are characteristically localized within the variable regions flanking the conserved phage core, situated between the core genes and the attachment sites. In contrast, 78.9% of ICE subunits carried tetracycline resistance genes, mainly *tet*(O)+*tet*(40) (*N* = 36) (data S1). In addition, phage-encoded surface proteins clustered within the Clade_Sag subgroup of SMphages (Fig. 1C), consistent with roles in adhesion and immune evasion (*27*). Together, these data establish phage-ICEs as composite tandems that serve as colocalized, complementary AMR reservoirs in clinically relevant streptococci.

### Phage-ICEs switch between excision and integration in response to stress

Chromosomally integrated MGEs alternate between excision and integration to balance vertical inheritance with horizontal spread (*30*). We quantified this equilibrium in *S. suis* isolates AKJ18 (phage-ICE orientation) and SC070731 (ICE-phage orientation) under basal and stress conditions (Fig. 2A and fig. S3A).

**Fig. 2.**
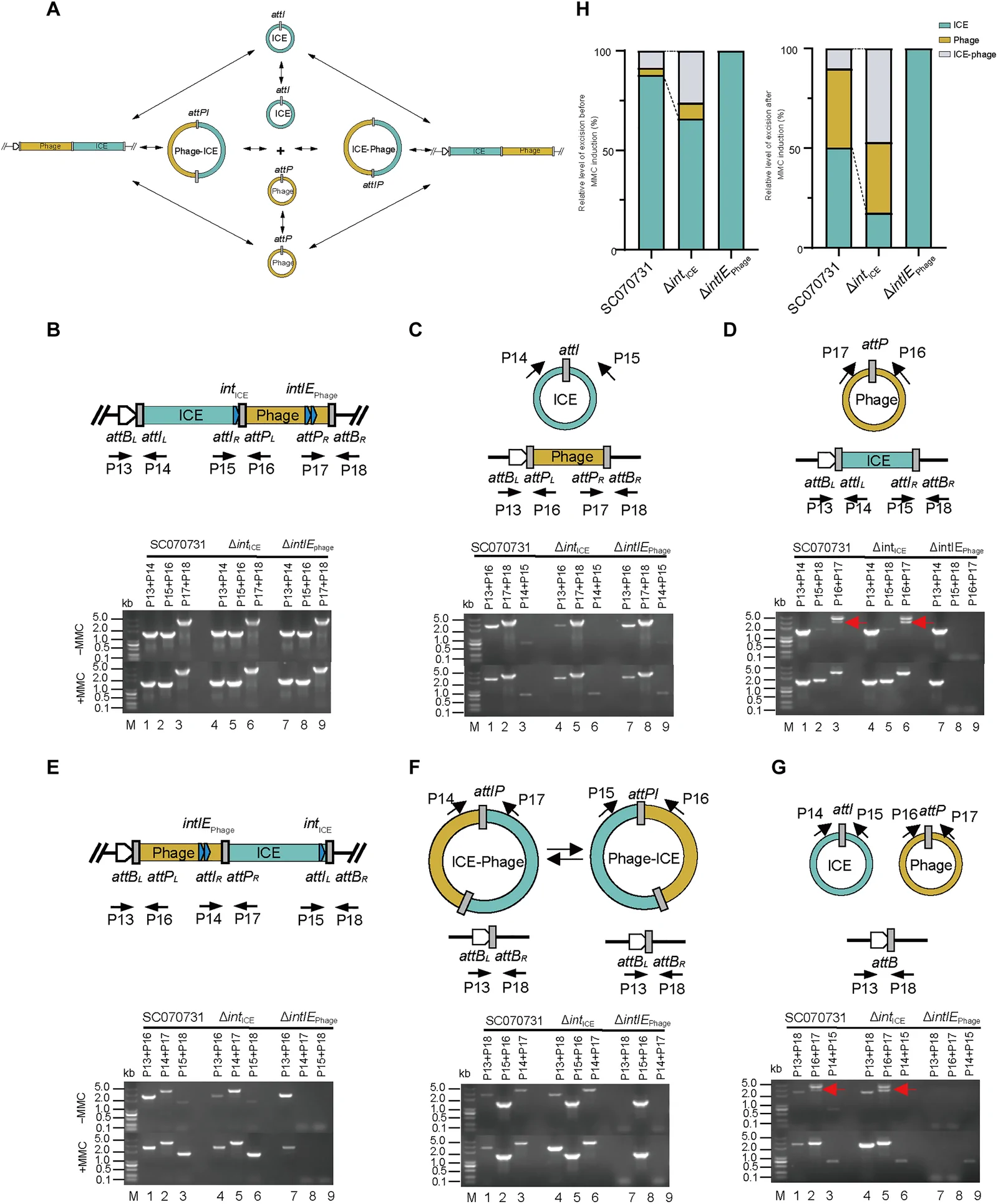
Excision-integration dynamics of phage-ICEs in *S. suis* SC070731. (**A**) Schematic of possible integrated and circularized states for phage, ICE, and tandem modules. (**B** to **G**) PCR detection of excision/circularization and reintegration products of phage, ICE, tandems (ICE-Phage/phage-ICE) in strains SC070731 (lanes 1 to 3), Δ*int*_ICE_ (lanes 4 to 6), and Δ*intIE*_Phage_ (lanes 7 to 9), with or without MMC. Genomic DNA (50 ng) was prepared from cultures induced for 1 hour with MMC (0 or 0.5 μg/ml) at OD_600_ ≈ 0.5 and used as the PCR template. M, DNA marker. (**H**) Quantitative analysis of excision frequencies for phage-only, ICE-only, and tandem products across indicated strains (*S. suis* SC070731, Δ*int*_Phage_, and Δ*int*_ICE_), based on grayscale intensity measurements. Note: The upper band observed with primers 17 to 16 [(D) and (G)] is a nonspecific amplification product; WGS confirmed the stable carriage of AMR genes within the phage module. The relative abundance of each excised form (ICE, phage, or tandem) was calculated by dividing the integrated density of its specific band by the total density of all detected excision bands within the same sample.

During routine growth, both strains maintained a dynamic balance: Phage excision was infrequent (AKJ18: 9.8%; SC070731: 3.4%), ICE excision more frequent (AKJ18: 80.1%; SC070731: 87.9%), and tandem excision rare (phage-ICE: 10.1%; ICE-phage: 8.7%) (Fig. 2, C, D, and F, and fig. S3, B to D). To determine whether this low frequency resulted from host cell death following phage excision, we monitored cell viability [colony-forming units (CFU)/ml] under both spontaneous and mitomycin C (MMC)–induced conditions. Only when induced by MMC for a long period of time will the bacteria undergo lysis and die. Under normal growth conditions, the number of viable bacteria gradually increases (fig. S3H), indicating that module excision in this system does not immediately trigger the lytic cycle or compromise host survival. This suggests that the measured frequencies reflect the intrinsic excision dynamics of the tandem modules rather than an artifact of differential cell mortality.

In addition, diagnostic polymerase chain reaction (PCR) detected all six predicted excision/reintegration intermediates in both SC070731 (Fig. 2, A to G, and table S1) and AKJ18 (fig. S3, B to G), indicating that spontaneous configuration switching is a conserved feature across different phage-ICE orientations.

MMC elicited orientation-specific responses. In SC070731, MMC increased phage excision 20.8-fold (*P* < 0.0001) and tandem excision 2.1-fold (*P* < 0.01), with preferential reintegration into the reverse phage-ICE orientation (Fig. 2, B to G). In AKJ18, MMC selectively promoted phage circularization (7.7-fold; *P* < 0.0001), whereas ICE and tandem forms remained integrated (fig. S3, B to D). These findings demonstrate that the structural arrangement of the tandem strongly dictates its response to environmental stress. This orientation-dependent modularity allows the elements to alternate between coordinated and independent excision dynamics, providing the tandem with regulatory flexibility to sense and adapt to SOS-stress signals.

To define genetic requirements, we constructed deletion mutants in SC070731. Loss of phage integrases IntIE_Phage_ (Δ*intIE*_Phage_) abolished excision of both phage and tandem modules even under MMC stress, whereas deletion of ICE integrase Int_ICE_ (Δ*int*_ICE_) reduced, but did not eliminate, ICE excision (Fig. 2H). These data implicate phage-encoded integrases as supplementary contributors to ICE excision and essential drivers of phage-ICE tandem dynamics, provided the foundation for the common dissemination of these components.

### Phage integrases promote phage-ICE excision and circularization

Phage-ICEs encode two integrase systems: IntIE_Phage_ (phage-derived) and Int_ICE_ (ICE-derived) (Fig. 3A). Sequence comparisons indicated recognition of distinct attachment (*att*) sites (fig. S4A), including an 8–base pair (bp) motif conserved in *Streptococcus* and a shorter GA dinucleotide previously misannotated in *S. pyogenes* (*27*, *31*).

**Fig. 3.**
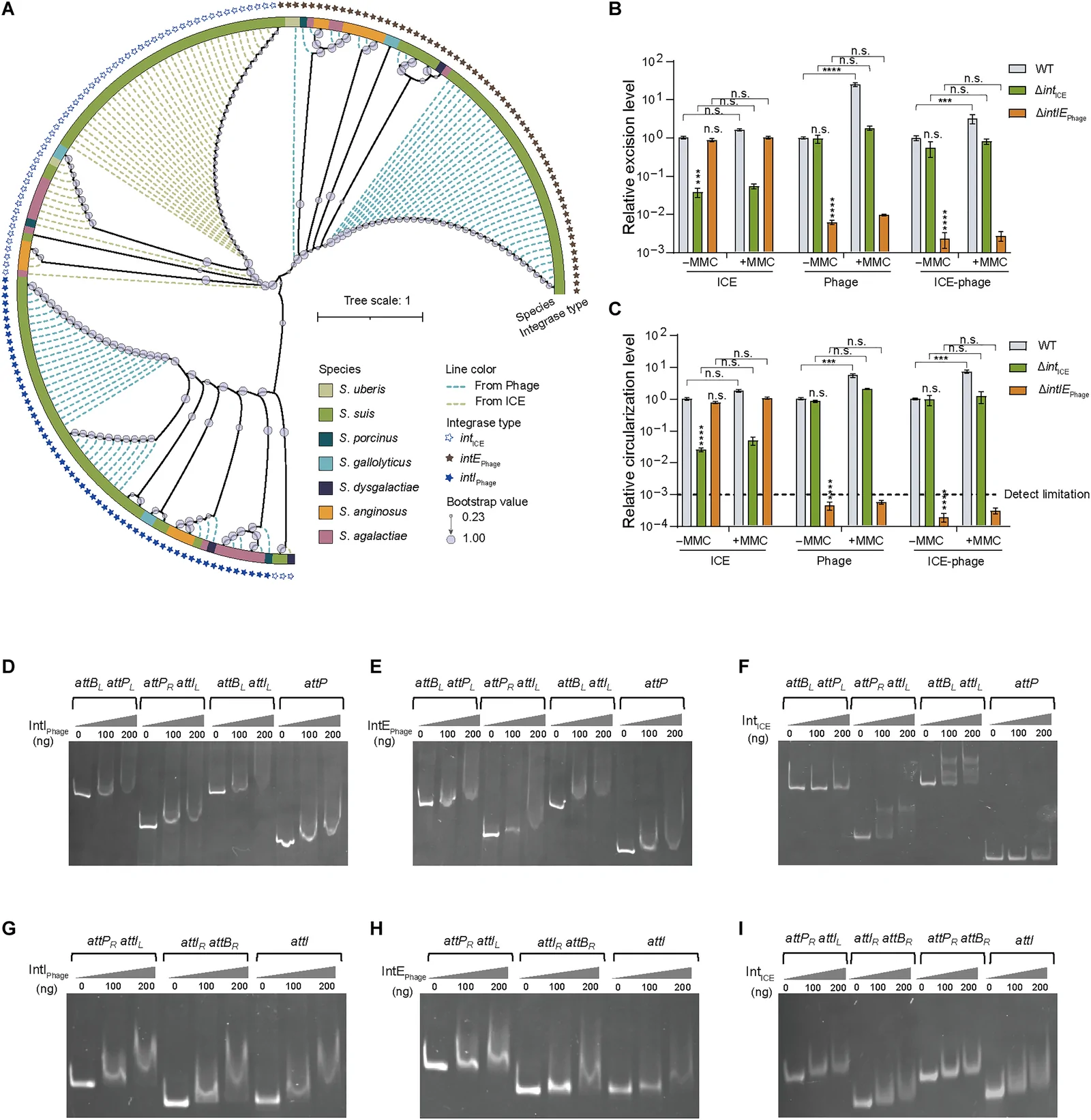
Phage integrases facilitate ICE and phage-ICE excision and circularization. (**A**) Phylogenetic analysis of IntI_Phage_, IntE_Phage_, and Int_ICE_ from 57 *Streptococcus* strains harboring phage-ICEs with complete boundaries. Representative clades are marked with blue, brown, and white stars, respectively; background shading indicates bacterial species. (**B** and **C**) Excision/circularization frequencies of ICE/phage/tandem structures in WT (SC070731), Δ*int*_Phage_, and Δ*int*_ICE_ strains, with and without MMC. (**D** to **I**) EMSA of IntI_Phage_, IntE_Phage_, and Int_ICE_ with their *att* site probes from *S. suis* AKJ18. IntI_Phage_ and IntE_Phage_ bind all *att* sequences tested, whereas Int_ICE_ binds its cognate *att* sites but not *attB_L_ attP_L_* and *attP.* “(−)”: no protein control. ***P < 0.001, ****P < 0.0001; n.s., not significant.

Quantitative PCR (qPCR) revealed distinct, asymmetric contributions of the integrases to excision and circularization—the intermediates required for conjugation. As expected, each integrase was indispensable for its cognate element: *ΔintIE*_Phage_ led to near-complete loss of phage excision/circularization, whereas *Δint*_ICE_ caused a marked but partial reduction in ICE excision (Fig. 3, B and C). Notably, IntIE_Phage_ also promoted circularization of ICE and phage-ICE tandems as ΔIntIE_Phage_ significantly reduced (but did not abolish) their excision efficiency. In contrast, ΔInt_ICE_ had little to no effect on phage or phage-ICE circularization, indicating a nonsymmetric division of labor. Thus, phage integrases both drive phage excision and provide cross-module support for ICE/tandem episome formation, expanding the pool of transferable intermediates. Even in the absence of phage integrase activity, phage-derived fragments disseminated via transformation and integrated through HR (see the next section), providing an alternative route when canonical circularization is impaired.

Electrophoretic mobility shift assay (EMSA) corroborated these findings. Purified IntI_Phage_ and IntE_Phage_ bound strongly to *att* sequences from phage, ICE, and phage-ICE junctions (Fig. 3, D, E, G, and H, and fig. S4), whereas Int_ICE_ did not detectably bind phage-derived *att* sequences under the same conditions (Fig. 3, F and I, and fig. S4). Together, the data support a three-tier model: (i) each integrase primarily governs its cognate element, (ii) phage integrases extend activity to ICE/tandem circularization, and (iii) ICE integrase exerts limited influence on phage/tandem modules. This asymmetry suggests that, in such phage-ICE tandems, the phage-encoded recombinase may act as a versatile driver for generating various circular intermediates. Whether this hierarchical interaction is a widespread regulatory strategy among diverse tandem MGEs remains an intriguing question for further investigation.

### Phage-ICEs exploit diverse HGT pathways for dissemination

In the absence of transduction-mediated dissemination for phage-ICE tandems, we examined whether they spread via conjugation or transformation through multiple routes—independent ICE transfer, phage transfer, and cotransfer—consistent with our previous finding that SMphages hijack the T4SS of helper ICE elements for intercellular transfer (*27*). Using *S. suis* AKJ18 (phage-ICE orientation) as the donor and P1/7RF as the recipient, we detected (i) conjugative transfer of ICE-only [ICE*Ssu*AKJ18_*rum*; (1.21 ± 2.09) × 10^−7^]; (ii) T4SS-mediated mobilization of the phage module in trans [Phage*Ssu*AKJ18_*rum*; (2.15 ± 1.00) × 10^−7^], confirmed to be independent of viral particle formation (see Materials and Methods); and (iii) conduction of the tandem [phage-ICE*Ssu*AKJ18_*rum*; (1.36 ± 1.12) × 10^−9^] (Fig. 4A). Tandem conduction occurred at >100-fold lower frequency than single-module transfer. This reduced efficiency likely reflects the combined effects of the significantly lower spontaneous excision frequency of the 130-kb tandem (as shown in Fig. 2) and the inherent physical constraints associated with the conjugative transfer of large DNA cargos (*32*). Integration at the *rum* locus was verified by pulsed-field gel electrophoresis (PFGE) and Southern blotting with *tet*(*O*) and *lsa*(*E*) probes (Fig. 4B and table S1).

**Fig. 4.**
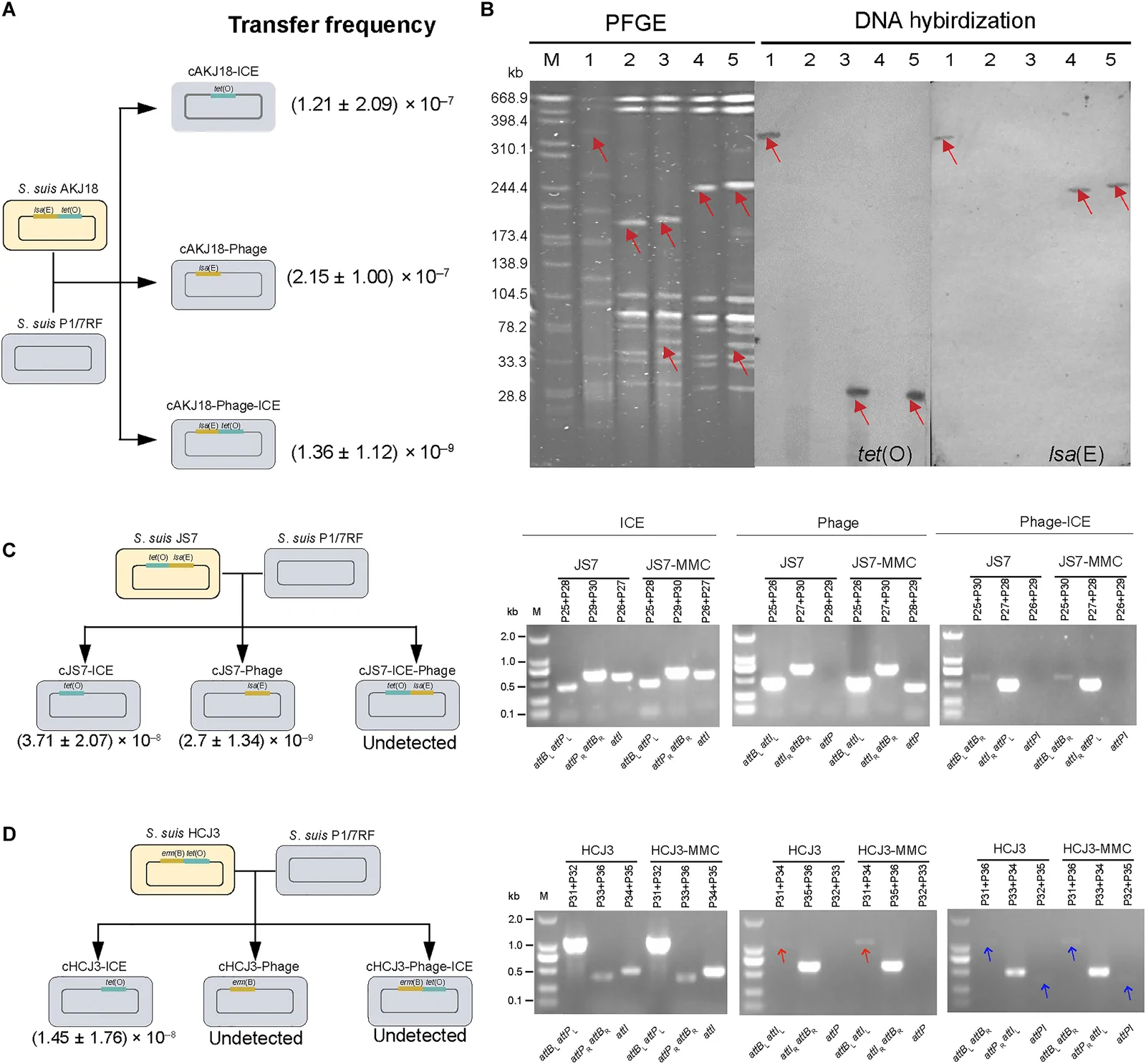
Diverse HGT pathways for ICE-phage dissemination. (**A**, **C**, and **D**, left) Schematics of conjugation assays. Donor and recipient cells are shaded pale yellow and gray, respectively; phage and ICE segments are shown as earthen yellow and blue bars. Donor: *S. suis* AKJ18, *S. suis* JS7 and *S. suis* HCJ3; recipient: *S. suis* P1/7RF. (**B**) PFGE and Southern blot analysis of transfer pairs. Lanes: M, size marker (*Salmonella* Braenderup H9812); 1 to 5, *S. suis* AKJ18, P1/7RF, cAKJ18_ICE, cAKJ18_Phage, and cAKJ18_Phage-ICE. Red arrows indicate shifted or novel bands hybridizing to ARG probes. [(C) and (D), right)] PCR detection of ICE, phage, and tandem excision/circularization in strains JS7 and HCJ3, with and without MMC induction. DNA extraction and lane notation as in Fig. 2.

Across 15 additional *S. suis* isolates (table S2), two further patterns emerged: (i) ICE conjugation with phage mobilization but no tandem conduction (e.g., JS7), likely due to structural constraints that render tandem excision extremely inefficient, with no detectable circular intermediates under our experimental conditions (Fig. 4C); and (ii) ICE-exclusive conjugation (e.g., HCJ3), where limited excision and weak circularization of phage or phage-ICE episomes prevented phage mobilization (Fig. 4D).

In HCJ3, with markedly reduced IntIE_Phage_ activity, phage excision was detectable but did not yield stable circular intermediates; weak tandem circularization signals were also insufficient for conjugation, and no phage- or tandem-containing transconjugants were recovered (Fig. 4D). By contrast, DNase I–free transformation yielded both phage- and phage-ICE–harboring transformants (Fig. 5, A and B, and table S1), indicating that transformation compensates when episomes form inefficiently. Thus, phage-ICEs use dual strategies: conjugation when circular intermediates are available and transformation as a contingency when episomalization is impaired.

**Fig. 5.**
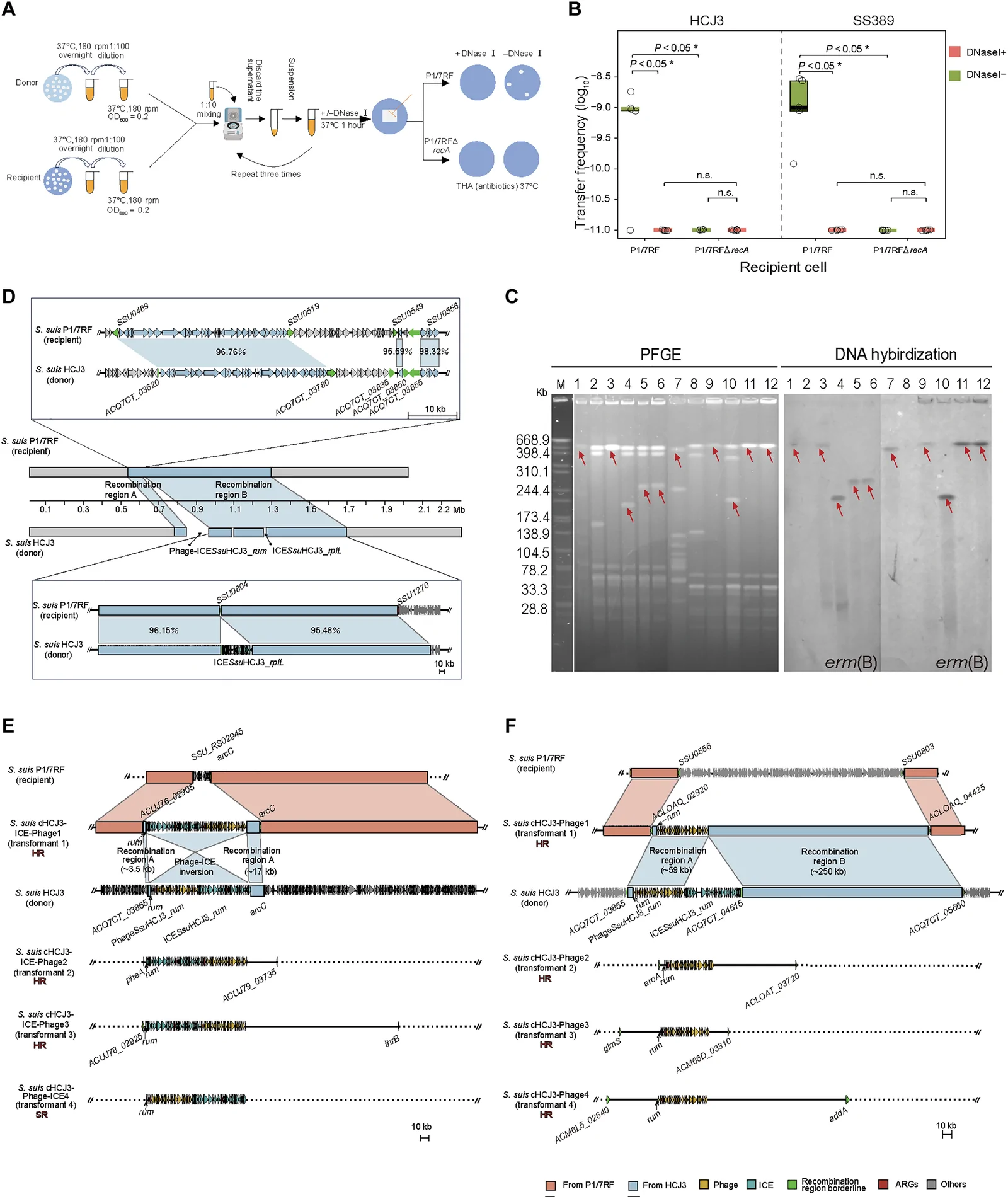
Heterogeneous integration of phage- and phage-ICE–derived sequences via HR. (**A**) Experimental workflow for HGT assays. (**B**) Transfer assays with and without DNase I. Deletion of *recA* in the recipient P1/7RF (P1/7RFΔ*recA*) abolished phage/phage-ICE transfer (*P* < 0.05; n.s., not significant). (**C**) PFGE and Southern blotting of transfer pairs. Lanes: M, marker; 1 to 12, *S. suis* HCJ3, P1/7RF, cHCJ3_Phage1–4, SS389, P1/7RF, and cSS389_Phage1–4. Red arrows indicate mobility shifts and probe-hybridizing fragments. (**D**) Genomic homology between *S. suis* HCJ3 and P1/7RF. Homologous regions are color coded. (**E** and **F**) Genomic comparison and integration patterns of phage-ICE (E) and phage-only (F) fragments. Light red/dashed lines: recipient genes; blue/solid lines: donor genes. Yellow: phage; mint green: ICE; emerald green: homology key genes; red: ARGs; gray: other genes; the position of “*rum*” is indicated by the arrow.

Beyond direct transfer, bidirectional mating of strains carrying single MGEs showed that SC070731ΔPhage (ICE-only donor) and cHCJ3_Phage1 (phage-only recipient) reconstituted a tandem element (Phage*Ssu*HCJ3-ICE*Ssu*SC070731_*rum*) at *rum* with a frequency of (1.25 ± 1.37) × 10^−9^ (Table 1). Reverse transfer failed, and reciprocal crosses with SS40AE (phage-only) and cAKJ18_ICE (ICE-only) yielded no transfer, indicating selective compatibility. These data demonstrate de novo tandem assembly via site-specific recombination (SR) between independent modules and motivate assessment of HR in broader structural diversification.

**Table 1.** Characteristics of the strains for which MGE successfully transferred. TET, tetracycline; ERY, erythromycin; TIA, tiamulin; CLI, clindamycin; FFC, florfenicol; GEN, gentamicin; LZD, linezolid. MICs in bold represent resistance to corresponding antimicrobials according to CLSI M100-ED29.

Donor	Transconjugant/transformant	MGE [AMR gene] transferred	Transfer frequency* (× 10^−9^)	MIC (mg/liter)						
				TET	ERY	TIA	CLI	FFC	GEN	LZD
Recipient *S. suis* P1/7RF				4	≤0.5	2	≤0.5	≤0.5	4	≤0.5
*S. suis* AKJ18		Phage-ICE*Ssu*AKJ18_*rum* [*erm*(B), *tet*(O), *lsa*(E), *lnu*(B), *aadE*, *sat4*, *aph(3ʹ)-III*]		**128**	**64**	**64**	**256**	≤0.5	2	≤0.5
	cAKJ18_ICE	ICE*Ssu*AKJ18_*rum* [*erm*(B), *tet*(O)]	**121 ± 209**	**128**	**≥256**	≤0.5	**128**	≤0.5	2	≤0.5
	cAKJ18_Phage	Phage*Ssu*AKJ18_*rum* [*lsa*(E), *lnu*(B), *aadE*, *sat4*, *aph(3ʹ)-III*]	**215 ± 100**	4	≤0.5	**32**	**128**	≤0.5	4	≤0.5
	cAKJ18_Phage-ICE	Phage-ICE*Ssu*AKJ18_*rum* [*erm*(B), *tet*(O), *lsa*(E), *lnu*(B), *aadE*, *sat4*, *aph(3ʹ)-III*]	1.36 ± 1.12	**64**	**≥256**	**16**	**128**	≤0.5	2	≤0.5
*S. suis* HCJ3		Phage-ICE*Ssu*HCJ3_*rum* [*erm*(B), *aadE*, *aac(6ʹ)-aph(2ʹ), tet*(O)]		**32**	**≥256**	0.5	**128**	≤0.5	**≥256**	≤0.5
	cHCJ3_ICE	ICE*Ssu*HCJ3_*rum* [*tet*(O)]	**215 ± 107**	**128**	≤0.5	0.25	0.25	≤0.5	4	≤0.5
	cHCJ3_ICE-Phage1	Phage-ICE*Ssu*HCJ3_*rum*-carrying fragment [*erm*(B), *aadE*, *aac(6ʹ)-aph(2ʹ), tet*(O)]	1.23 ± 1.25	**128**	**≥256**	0.5	**128**	≤0.5	**≥256**	≤0.5
	cHCJ3_ICE-Phage2		1.81 ± 1.15	**128**	**≥256**	0.5	**64**	≤0.5	**256**	≤0.5
	cHCJ3_ICE-Phage3		1.55 ± 2.21	**128**	**≥256**	0.5	**128**	≤0.5	**≥256**	≤0.5
	cHCJ3_Phage-ICE4	Phage-ICE*Ssu*HCJ3_*rum* [*erm*(B), *aadE*, *aac(6ʹ)-aph(2ʹ), tet*(O)]	1.49 ± 2.37	**128**	**≥256**	0.5	**128**	≤0.5	**≥256**	≤0.5
	cHCJ3_Phage1	Phage*Ssu*HCJ3_*rum*-carrying fragment [*erm*(B), *aadE*, *aac(6ʹ)-aph(2ʹ)*]	1.23 ± 1.25	1	**≥256**	0.5	**64**	≤0.5	**≥256**	≤0.5
	cHCJ3_Phage2		1.81 ± 1.15	1	**≥256**	0.5	**128**	≤0.5	**≥256**	≤0.5
	cHCJ3_Phage3		1.55 ± 2.21	1	**≥256**	0.5	**64**	≤0.5	**≥256**	≤0.5
	cHCJ3_Phage4		1.49 ± 2.37	1	**≥256**	≤0.25	**128**	≤0.5	**≥256**	≤0.5
*S. suis* SS389		Phage*Ssu*SS389*_rum* [*erm*(B)*, optrA, tet*(O)*, tet*(40)*, aadE, aac(6ʹ)-aph(2ʹ)*]		**64**	**≥256**	0.5	**128**	**16**	**256**	**4**
	cSS389_Phage1	Phage*Ssu*SS389_*rum*-carrying fragment [*erm*(B)*, optrA, tet*(O)*, tet*(40)*, aadE, aac(6ʹ)-aph(2ʹ)*]	2.98 ± 2.47	**32**	**≥256**	1	**64**	**16**	**256**	**4**
	cSS389_Phage2		2.74 ± 1.59	**32**	**≥256**	1	**128**	**16**	**256**	**4**
	cSS389_Phage3		3.12 ± 1.62	**32**	**≥256**	0.5	**64**	**16**	**256**	**4**
	cSS389_Phage4		3.55 ± 2.27	**32**	**≥256**	0.5	**128**	**16**	**256**	**4**
*S. suis* JS7		ICE-Phage*Ssu*JS7_*rum* [*optrA*, *erm*(B), *tet*(O), *catA9*, *lsa*(E), *lnu*(B), *aadE*, *ant(9)-I*]		**128**	**≥256**	**32**	**64**	**16**	2	**4**
	cJS7_ICE	ICE*Ssu*JS7_*rum* [*optrA*, *erm*(B), *tet*(O), *catA9*]	**37.1 ± 20.7**	**128**	**≥256**	0.5	≤0.5	**16**	2	**4**
	cJS7_Phage	Phage*Ssu*JS7_*rum* [*lsa*(E), *lnu*(B), *aadE*, *ant(9)-I*]	2.7 ± 1.34	1	≤0.5	**32**	**64**	≤0.5	2	≤0.5
Recipient *S. suis* cHCJ3_Phage1†		Phage*Ssu*HCJ3_*rum* [*erm*(B), *aadE*, *aac(6ʹ)-aph(2ʹ)*]		2	**≥256**	≤0.5	**64**	≤0.5	**≥256**	≤0.5
*S. suis* SC070731ΔPhage_*rum*		ICE*Ssu*SC070731_*rum* [*tet*(O), *tet*(40)]		**128**	≤0.5	2	≤0.5	≤0.5	4	≤0.5
	P1/7RF::Phage*’*_ICE	Phage*Ssu*HCJ3-ICE*Ssu*SC070731_*rum* [*erm*(B), *aadE*, *aac(6ʹ)-aph(2ʹ)*, *tet*(O), *tet*(40)]	1.25 ± 1.37	**128**	**≥256**	≤0.5	**64**	≤0.5	**≥256**	≤0.5

* Transfer frequency was calculated on the basis of the number of observed transconjugants divided by the initial number of donors in each assay of three duplicates.

† Recipient *S. suis* cHCJ3_Phage1 was derived from P1/7RF by acquisition of Phage*Ssu*HCJ3_*rum* from *S. suis* HCJ3.

### HR drives flexible, length-variable integration of phage-ICE fragments

To assess HR’s contribution, we performed DNase I–free filter mating assays using donors *S. suis* HCJ3 (phage-ICE_*rum*, with nearly abolished IntIE_Phage_ activity) or SS389 (Phage_*rum*, ICE-negative) and selected for transformants carrying integrated phage-ICE or phage fragments (Fig. 5A). Whole-genome sequencing (WGS) indicated predominant HR-mediated integration rather than integrase-mediated SR (Fig. 5, B and C). For phage-ICE cotransfers from HCJ3, integration was largely HR driven with occasional SR events, suggesting residual integrase activity in specific contexts (Fig. 5, B and C). Deletion of *recA* in recipient P1/7RF abolished detectable phage/phage-ICE transfer (Fig. 5B), establishing HR as essential when site-specific integrases are nonfunctional.

Comparative genomic analysis revealed extensive donor-recipient homology. In HCJ3, a 70-kb region upstream of *rum* (98.32, 95.59, and 96.76% identity) and a 750-kb region downstream of *glf* (96.15 and 95.48%) provided substrates for recombination (Fig. 5D). HR-mediated recombinants (cHCJ3_Phage–ICE1–3; cHCJ3_Phage1–4) consistently replaced the recipient *rum* locus but varied in flanking tract lengths and orientations, including inversions of phage and ICE modules (Fig. 5E). The observed inversions in these recombinants reflect the intrinsic mobility and reintegration flexibility of the phage-ICE tandem, consistent with the spontaneous excision and circularization dynamics characterized in Fig. 2. For cHCJ3_Phage–ICE1–3, upstream donor tracts were 3.5, 2.7, and 1.4 kb with downstream tracts of 17.2, 36.8, and 196.3 kb, respectively (Fig. 5E). For cHCJ3_Phage1–2, upstream tracts were ∼5.5 kb with downstream tracts of ∼250 and ∼92 kb, whereas cHCJ3_Phage3–4 carried longer upstream tracts (∼43 and ∼54 kb) that disrupted the capsular polysaccharide (*cps*) locus, with downstream tracts of ∼19 and ∼155 kb (Fig. 5F). Donors from SS389, reflecting greater sequence divergence, yielded multisite replacements and large inversions (fig. S5, A to E).

Collectively, these data show that phage-carrying elements—including phage-ICEs—integrate via HR with remarkable structural flexibility, producing length-variable rearrangements. Beyond AMR acquisition (Table 1), underscoring their potential to reshape genome architecture in streptococci.

### HR enhances host genomic plasticity and stress adaptation

To examine the broader phenotypic consequences of the large-scale chromosomal replacements observed in HR-derived transformations, we compared HR- versus SR-derived transconjugants/transformations. Given that MMC treatment increased excision and circularization of these elements (Figs. 2 and 4 and fig. S3), indicating that environmental stress can influence their dynamics, we further asked whether recombinants generated after transfer might exhibit altered fitness under stress conditions. We hypothesized that such extensive genomic “rewiring” might confer fitness advantages beyond antibiotic resistance, particularly in response to environmental challenges encountered during infection. Six HR-derived strains were analyzed—four from phage-only transfer (cPhage1 and cPhage3 from *S. suis* HCJ3; cPhage2 and cPhage4 from SS389) and two from phage-ICE cotransfer (cICE-Phage2 and cICE-Phage3 from HCJ3)—alongside two SR-derived strains (cPhage from AKJ18; cPhage-ICE4 from HCJ3).

In head-to-head competition under nonstress conditions, HR-derived transconjugants consistently outcompeted the recipient P1/7RF (Fig. 6A). Across stressors [ultraviolet (UV), MMC, and H_2_O_2_], only H_2_O_2_ produced clear differences: HR strains showed significantly smaller inhibition zones (Fig. 6B). SR-derived strains resembled the recipient, indicating that oxidative resilience is associated with HR-mediated integration. Because UV and MMC showed no HR-SR differences, subsequent analyses focused on oxidative stress. Genomic analyses excluded direct acquisition of canonical oxidative stress genes within recombined regions; these regions primarily encode core metabolic enzymes and conserved transcription factors common to both donor and recipient. Two HR transconjugants (cPhage2 and cPhage4) also integrated outside the *rum* locus yet retained fitness gains, arguing against insertion-site artifacts. These observations support large-scale chromosomal replacement and regulatory rewiring as key outcomes of HR.

**Fig. 6.**
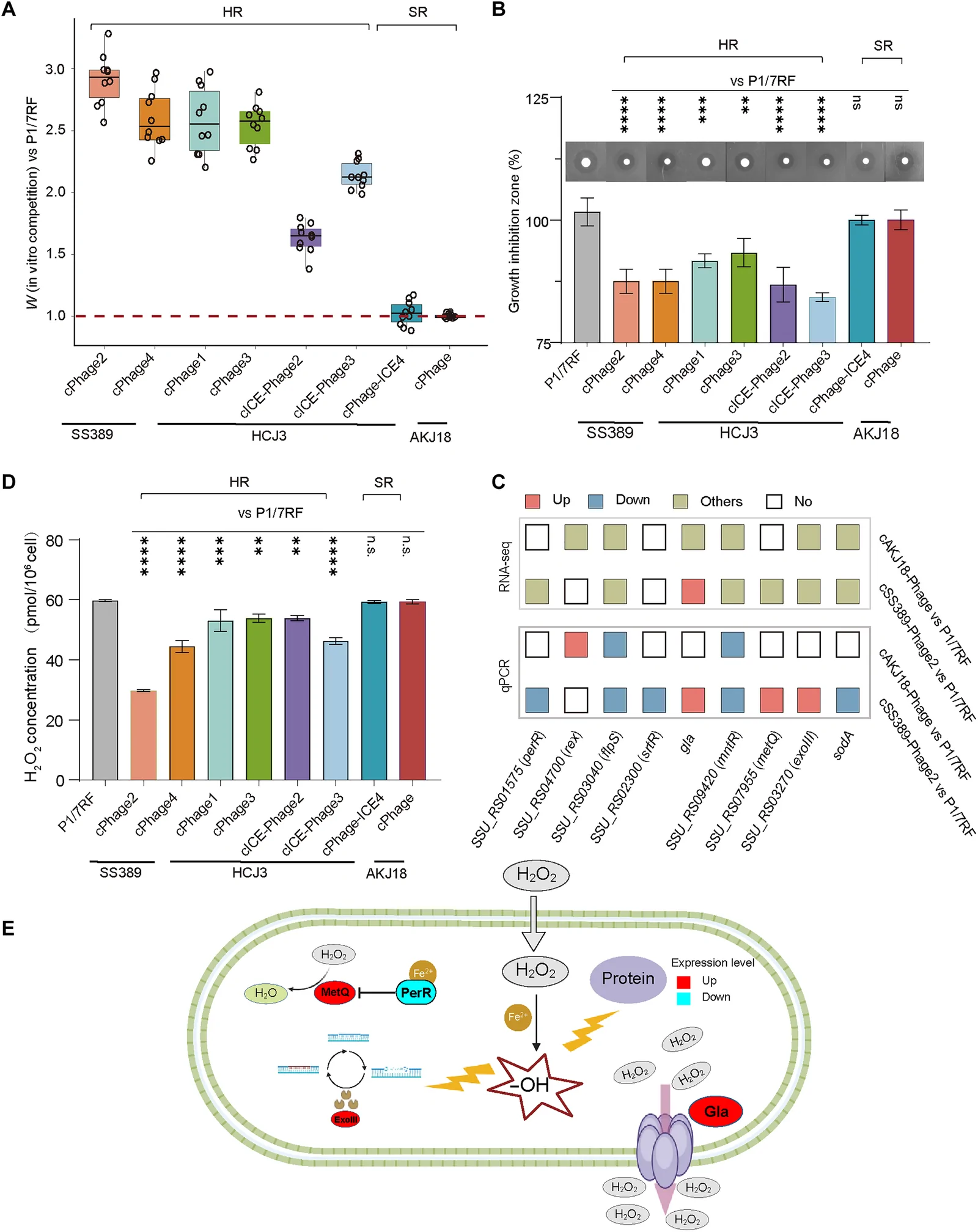
HR enhances host bacterial fitness and oxidative stress tolerance. (**A**) Competitive fitness (*W*) of eight selected transconjugants/transformants obtained via HR or SR versus recipient P1/7RF. Data are mean ± SD (*n* = 10). Abbreviations: HR, homologous recombination; SR, site-specific recombination. Statistical significance: **P < 0.01, ***P < 0.001, and ****P < 0.0001; n.s., not significant. (**B**) H_2_O_2_ sensitivity assayed by disk diffusion. Zones of growth inhibition are shown for the recipient strain *S. suis* P1/7RF and transconjugants/transformants. (**C**) Transcriptomic (RNA-seq) and qPCR validation of H_2_O_2_ stress response in P1/7RF, cSS389_Phage2 (HR), and cAKJ18_Phage (SR) after 25-min exposure. Red: up-regulated (log_2_FC ≥ 1, *P*_adj_ ≤ 0.05); blue: down-regulated (log_2_FC ≤ −1, *P*_adj_ ≤ 0.05); yellow: nonchanging; white: not significant. (**D**) Intracellular H_2_O_2_ levels after 25-min treatment with 25 mM H_2_O_2_. (**E**) Model of the H_2_O_2_ stress response in HR-integrated recombinants versus recipient (*84*). Red and blue indicate up-regulated and down-regulated genes, respectively.

Transcriptomic profiling under H_2_O_2_ stress revealed coherent signatures in genes that promote or repress peroxide responses. Positive effectors—*gla* (aquaporin-mediated peroxide efflux), *metQ* (peroxide detoxification), and *exoIII* (repair of peroxide-induced ssDNA breaks)—were consistently up-regulated, whereas *perR*, a negative regulator of antioxidant systems, was down-regulated. Additional modulators (*rex*, *flpS*, and *mntR*) showed concordant patterns (Fig. 6C and fig. S6, A to C). qPCR across six independent HR-derived transconjugants validated these trends for *gla*, *perR*, *rex*, *flpS*, *mntR*, and *exoIII* (fig. S6D). Notably, *gla* showed the strongest induction [|log_2_ fold change| (|log_2_FC|) ≥ 1, adjusted *P* ≤ 0.05]. In line with these transcriptional shifts, HR transconjugants accumulated significantly less intracellular H_2_O_2_ than the recipient after challenge (Fig. 6D). Together, the data indicate that HR reshapes genomic context to enable context-dependent activation of stress-response pathways, enhancing detoxification capacity and oxidative stress resilience (Fig. 6E).

### Phage-ICEs are evolutionarily widespread and functionally adapted

To map diversity and distribution, we developed a high-throughput pipeline and analyzed 314,818 microbial genomes from the NCBI RefSeq database (accessed December 2023). We identified 541,373 phages, 137,734 ICEs, and initially identified 11,012 putative tandems (∼3.5%), but many were incomplete, highly degraded, or lacked clear boundaries. After manual curation, 590 high-confidence previously unidentified tandems were retained, which, together with the *Streptococcus* tandems above, yielded a set of 612 unique elements for downstream analysis (fig. S7A and data S2).

Phage-ICEs were detected across seven phyla and 135 species, most prevalent in *Pseudomonas* (*N* = 221), *Streptococcus* (*N* = 108), *Staphylococcus* (*N* = 54), *Enterococcus* (*N* = 35), and *Mycobacteroides* (*N* = 28) (Fig. 7, A and B). These genera include major ESKAPE/MDR pathogens—carbapenem-resistant *P. aeruginosa* (CRPA), methicillin-resistant *S. aureus* (MRSA), and vancomycin-resistant enterococci (VRE)—underscoring clinical relevance. Most elements formed tandems (phage-ICE or ICE-phage; 72.5%), accounting for their greater lengths relative to single phage or ICE sequences (Fig. 7C and figs. S7B and S8). We also identified previously unrecognized structures: ICE::Phage (ICEs inserted into phage genomes, e.g., *S. aureus* M3601) and Phage::ICE (phages integrated within ICE modules, e.g., *Comamonas thiooxydans* ZM19) (Fig. 7C). Detailed mapping of these nested configurations revealed 18 distinct insertion loci; although many target hypothetical proteins or intergenic regions, several elements specifically use conserved functional genes such as the protease gene *clpP* and AT-rich intergenic sequences as integration hotspots (data S3). Integration site mapping of the broader dataset revealed 71 *att* sites; the most frequent included tRNA-Gly (32.1%), tRNA-Leu (5.5%), tRNA-Arg (4.0%), and *rlmD* (4.7%), with lineage-specific preferences (*P. aeruginosa*, tRNA-Gly; *S. suis*, *rum*) (Fig. 7B and fig. S9).

**Fig. 7.**
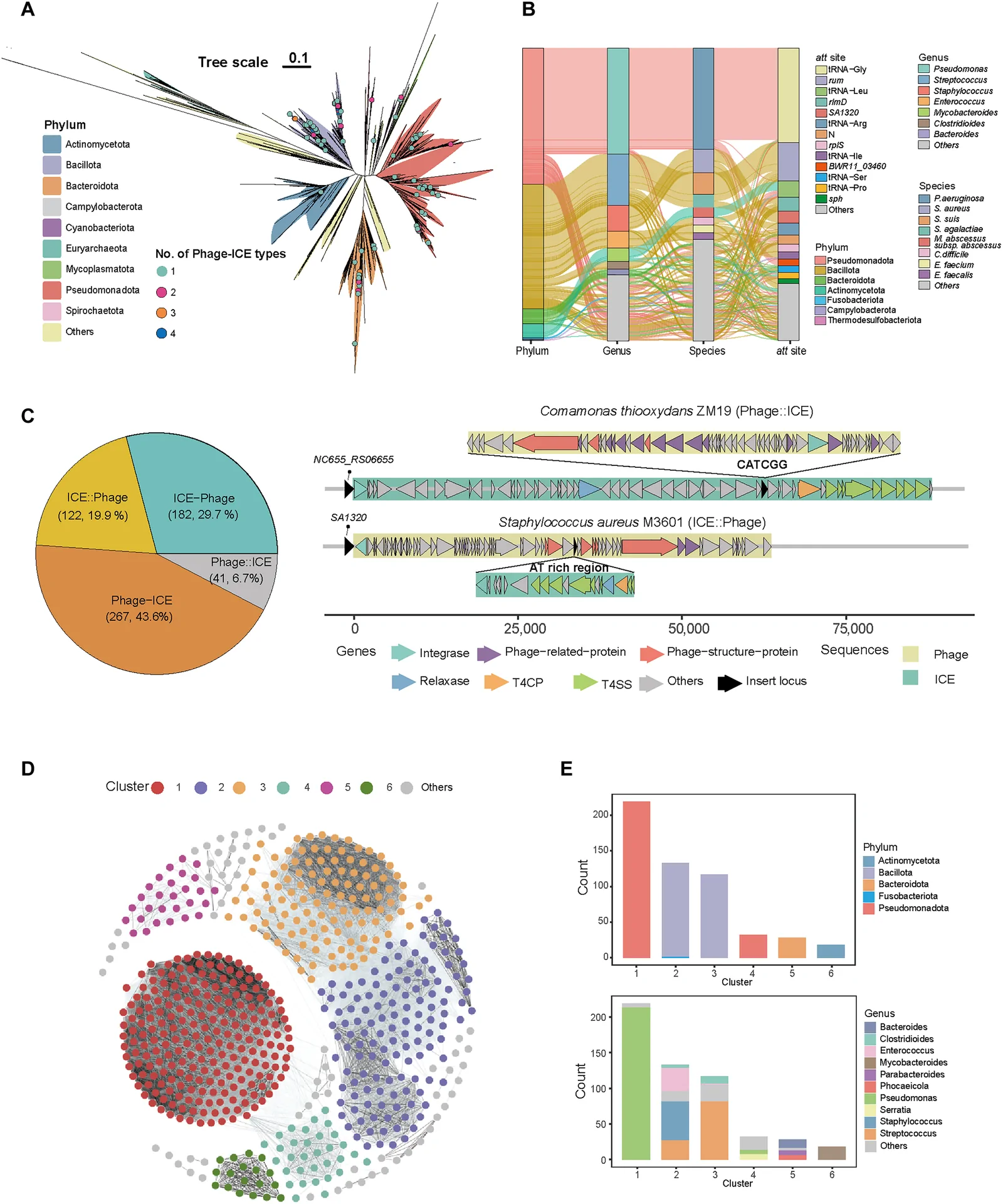
Global distribution and community structure of phage-ICEs. (**A**) 16*S* rRNA phylogeny of 19,083 bacterial and archaeal genomes; branches are color coded by phylum. Nodes indicate counts of phage-ICE types (ICE-phage, phage-ICE, ICE::Phage, and Phage::ICE). (**B**) Flowchart summarizing *att* site usage across species. (**C**) (Left) Counts of phage-ICE structural types. Phage::ICE denotes phage insertion within ICE; ICE::Phage denotes ICE insertion within phage; ICE-phage and phage-ICE denote tandems with ICE left/phage right and vice versa. (Right) Schematics of genomic regions encoding Phage::ICE and ICE::Phage. (**D**) Sequence similarity network of phage-ICEs. Nodes represent individual phage-ICEs; edges indicate the wGRR. Colors denote cluster assignments (Leiden clustering). (**E**) Host distribution across phyla and genera within clusters (top 6 shown).

Weighted gene repertoire relatedness (wGRR) and Leiden clustering partitioned the phage-ICEs into 39 communities (Fig. 7D). Although many communities aligned with phylum-level groupings, several were genus-heterogeneous (Fig. 7E). Functional annotation showed clear lineage-specific signatures: a *Pseudomonas*-dominant cluster encoded *tssH* (type VI secretion) together with *bla*_VEB_; a *Staphylococcus*/*Enterococcus*/*Streptococcus* cluster was enriched for carbohydrate-active enzymes CAZymes; GH23 and GH73) plus adhesion/biofilm genes (*ecbA/fss3*); a *Streptococcus*-dominant cluster carried dense AMR repertoires [*mef*(A), *msr*(D), *aph(3′)-III*, *sat*, *aadE*, *erm*(B), *tet*(O), and *tet*(40)[ (fig. S7, C and D, and data S2).

Overall, phage-ICEs are widespread, structurally diverse, and functionally specialized. Their lineage-specific cargo—including AMR determinants, virulence factors, and metabolic functions—supports a model in which phage-ICEs provide adaptable genetic scaffolds that facilitate the spread of clinically important traits.

## DISCUSSION

MGEs are central drivers of bacterial adaptation and evolution (*10*, *29*), yet how physically linked MGEs cooperate remains incompletely defined (*33*). Here, we delineate phage-ICE tandems as modular elements formed by site-specific accretion (*24*). By integrating at conserved chromosomal hotspots, these tandems create stable scaffolds that colocalize AMR and virulence determinants and facilitate their horizontal movement across bacterial hosts.

Our comparative genomics shows that phage-ICEs are not confined to streptococci but span seven phyla and 135 species, including ESKAPE pathogens such as *P. aeruginosa*, *S. aureus*, and *E. faecalis* (Fig. 7A and data S2). Their broad taxonomic range is consistent with modular recombination enabling movement across phylogenetic boundaries (*10*, *34*, *35*). We identified at least 71 integration sites (Fig. 7B and data S2), with recurring hotspots (e.g., *rlmD*, tRNA-Gly, and *rum*), suggesting selection for loci that afford vertical stability with limited host cost (*36*). Lineage-specific site use—for example, *rum* in *S. suis* and tRNA-Gly in *P. aeruginosa*—implicates integration specificity as a determinant of host range and cargo retention (*37*, *38*).

Cargo composition further supports functional specialization. Streptococcal phage-ICEs are enriched for AMR determinants [*tet*(O), *erm*(B), *mef*(A), and *msr*(D)], concordant with surveillance links to macrolide and tetracycline resistance (*39*). In contrast, staphylococcal and enterococcal clusters carry CAZymes and adhesion genes, consistent with traits that support persistence on biotic surfaces (*40*). *Pseudomonas*-associated elements often encode both virulence (e.g., *tssH*) and β-lactam resistance (*bla*_VEB_) (*41*, *42*). The cocarriage of resistance and virulence within transferable scaffolds underscores the potential of phage-ICEs to intensify clinical risk (*43*).

Mechanistically, our data assign a central role to phage-encoded integrases in generating transferable intermediates. IntIE_Phage_ is necessary for phage excision/circularization and also enhances circularization of ICEs and phage-ICE tandems, whereas the ICE integrase has limited impact beyond its cognate element (Fig. 2, C, D, F, and G). Although the phage provides the excision machinery, the ICE drives dissemination via conjugation. This dynamic suggests that the prophage acts as a “hitchhiker” (*7*), exploiting the ICE’s conjugative apparatus for conduction (*13*). This distinctive arrangement contrasts with phage-plasmids where both replication and transfer modules are often fused into a single evolutionary unit (*19*, *20*); here, the elements retain distinct identities but function interdependently due to their tandem arrangement. The mobilization of the resulting episomes via ICE-encoded conjugation establishes a modular division of labor, where phage integrases generate the substrates for transfer and the ICE machinery drives dissemination. Although this cooperative modularity echoes the functional complementation seen in multifaceted MGEs like phage-plasmids (*20*, *44*), it represents a more plastic and potentially transient interaction between independent integrative elements. Excision-integration equilibria respond to external cues, MMC exposure produced orientation-specific induction (Fig. 2H), indicating that transfer competence is environmentally tunable.

Phage-ICE tandems also exploit multiple HGT routes (*45*, *46*). Depending on configuration and compatibility, transfer occurs via ICE-only conjugation, phage mobilization, tandem conduction, phage transformation, and tandem cotransformation (Figs. 4, A and B, and 5, A and B). This versatility mirrors the increasingly blurred boundaries between traditional conjugation and transduction observed in other complex MGE systems (*47*). We found that tandem cotransfer occurs at a significantly lower frequency than single-module mobilization—likely reflecting the metabolic and biophysical burden of moving larger genomic payloads—whereas the observed partner specificity resembles the modular host-range constraints documented in other multifaceted elements (*21*, *47*, *48*). Such modularity may bias dissemination toward specific selective pressures (e.g., ICE modules under antibiotic exposure versus phage modules during host-cell interactions), consistent with the specialized ecological roles proposed for diverse MGE arrays (*27*, *49*–*51*).

Critically, mobility persists when integrase-mediated circularization is ineffective (Fig. 4D). Phage-ICEs (or their fragments) disseminate via transformation coupled to RecA-dependent HR, generating length-variable insertions and structural rearrangements (Fig. 5, E and F). HR-derived transconjugants retained mobility and displayed enhanced tolerance to H_2_O_2_, associated with transcriptional rewiring (up-regulation of *gla*, *metQ*, and *exoIII*; reduced *perR*) and reduced intracellular peroxide (Fig. 6 and fig. S6). Thus, HR functions both as a contingency pathway for transfer and as a source of functional diversification, in line with broader roles of recombination in bacterial evolution (*52*–*55*).

In summary, phage-ICE tandems constitute a widespread class of composite MGEs that link excision, transfer, and recombination to maintain mobility and remodel bacterial genomes. Their functional organization—phage integrases producing transferable episomes, ICE conjugation mediating spread, and HR providing a compensatory, diversification-prone route—positions phage-ICE tandems as potential accelerators of adaptation and AMR dissemination. These features nominate discrete intervention points, including inhibition of integrase activity, destabilization of tandem architectures, and restriction of HR-mediated integration, to limit the spread and evolutionary potential of high-risk pathogens.

## MATERIALS AND METHODS

### Experimental design

This study aimed to elucidate how phage-ICE tandem elements interactions and recombination modes shape bacterial adaptation and AMR dissemination. All experimental procedures are described in the corresponding figure legends, including the number of biological replicates, statistical tests, and exact *P* values. Comparative genomics, conjugation assays, and stress-response experiments were performed in *S. suis* to evaluate integrase-mediated excision, ICE-driven cotransfer, and HR-dependent integration. RNA sequencing (RNA-seq) and qPCR analyses identified antioxidant genes associated with enhanced oxidative tolerance in HR-derived strains. All assays were independently repeated at least three times, and no data points were excluded as outliers. Unless otherwise stated, all experiments were successfully replicated with biologically independent samples.

### Bacterial strains and growth conditions

All strains and plasmids used in experiments are listed in table S2. *Streptococcus* strains were cultured in Todd-Hewitt broth (THB) or on THB agar plates supplemented with 5% (v/v) newborn calf serum and incubated overnight at 37°C. *Escherichia coli* and *Salmonella enterica* serovar Braenderup strains were grown in Luria-Bertani (LB) broth or on LB agar plates at 37°C. Antibiotics were used at the following concentrations when required: rifampicin (100 μg/ml), fusidic acid (100 μg/ml), tetracycline (8 μg/ml), tiamulin (8 μg/ml), erythromycin (50 μg/ml), spectinomycin (100 μg/ml), ampicillin (100 μg/ml), MMC (0.5 μg/ml), or H_2_O_2_ (25 mM).

### Identification of phage-ICE tandem elements

Putative phage-ICE tandem elements were identified from *Streptococcus* genomes by screening for signature integrase genes from known *S. suis* SC070731 tandem element (*56*), using BLASTN against the GenBank nr/nt and WGS databases (accessed October 2023), with thresholds of ≥50% identity and query coverage. Contigs containing both phage and ICE integrase genes were further analyzed for conserved phage modules (replication, modification, packaging, morphogenesis, and lysis) and ICE backbone genes. Attachment sites (*attL*, *attR*, and *attM*) and AMR genes were annotated. A total of 114 candidate elements were identified, with 57 complete tandem structures retained from seven *Streptococcus* species.

### Whole-genome sequencing

Bacterial genomic DNA was extracted using the E.Z.N.A. Bacterial DNA Kit (Omega Bio-Tek) following the manufacturer’s recommendations. Complete genomes of the donors and transconjugants in transfer assays were further subjected to long-read sequencing by using a PacBio RSII System (Pacific Biosciences, USA). Complete genomes were assembled preliminarily with SMRT Link v.5.0.1 with PacBio data and were further corrected with WGS Illumina data.

### Bioinformatic analysis

First, we extracted the Phage-ICE genes, as well as the ICE core genes (*virB4*, *virD4*, *relaxase*, and *int*_ICE_) and the Phage core genes (*orf13-55* and *intIE*_Phage_) from 57 *Streptococcus* genomes containing tandem elements for subsequent phylogenetic tree construction.

For the phylogenomic analysis of the phage-ICE tandems, OrthoFinder (v2.5.4) (*57*) was used to infer orthologous groups. Single-copy orthologous genes were individually aligned using Clustal Omega (v1.2.3), and the resulting alignments were concatenated to construct a supermatrix. Ambiguously aligned sites were removed using trimAl (v1.4.rev15) (*58*) with the “-automated1” option. Last, a maximum likelihood (ML) tree was reconstructed using IQ-TREE (v2.1.4) (*59*) with 1000 ultrafast bootstrap replicates, and the best-fit substitution model was automatically selected by ModelFinder (*60*).

For single-gene phylogenetic analysis (e.g., *virB4*, *virD4*, *relaxase*, *integrase*, etc.), the DNA sequences were aligned using the MEGA 11 (*61*). All trees were aesthetically refined using ITOL (*62*, *63*). Genomic comparisons were generated with Easyfig v2.2.2. (*64*).

### Antimicrobial susceptibility testing

Minimum inhibitory concentrations (MICs) were determined using a broth microdilution method for antimicrobial agents commonly used in veterinary and human medicine (Table 1), following CLSI guidelines (M100-ED29) (*65*). *S. aureus* ATCC 29213 was used for quality control.

### Genetic manipulation of bacterial strains

Deletion mutants (e.g., *ΔintIE*_Phage_, *Δint*_ICE_, and *ΔrecA*) were constructed using the temperature-sensitive plasmid pSET4s (*66*). Upstream and downstream fragments of target genes were amplified using specific primers (table S1), fused into linearized pSET4s via In-Fusion cloning, and introduced into *E. coli* DH5α before natural transformation into *S. suis*. Mutants were selected on spectinomycin and verified by PCR and sequencing.

### DNA extraction and excision/circularization detection

Bacterial genomic DNA was extracted using the E.Z.N.A. Bacterial DNA Kit (Omega Bio-Tek), according to the manufacturer’s instructions. Overnight bacterial cultures were diluted 1:100 into fresh THB and grown at 37°C with shaking at 200 rpm, until OD_600_ (optical density at 600 nm) = 0.5. Cultures were treated without or with MMC (0.5 μg/ml, final concentration) for 30 min, before bacterial DNA extraction. Total DNA was extracted according to the instructions of the kit. DNA concentration was measured using NanoDrop 2000 (Thermo Fisher Scientific, USA) and adjusted to same concentration as the PCR templates. PCR amplification was used to detect changes in ICE/phage excision and circularization in strain.

### Densitometric analysis of excision products

Comparative analysis of excision configurations in wild-type (WT) and mutant strains (Fig. 2H) were imaged using ImageJ (v1.8.0). The relative abundance of each excised form (ICE, Phage, or Tandem) was calculated by dividing the integrated density of its specific band by the total density of all detected excision bands within the same sample. This ratio represents the relative distribution of excision events across the different modules under the tested conditions.

### qPCR for excision/circularization frequencies and expression analysis

The relative levels of excision and circularization were quantified by qPCR using a modified protocol based on ref. (*67*). All qPCR primers are listed in table S1. To assess the dynamics in WT (SC070731) strains and their mutants, three primer pairs (qSCP1/qSCP4, qSCP3/qSCP6, and qSCP1/qSCP6) were designed to span the *attB* chromosomal junctions remaining after the excision of the ICE, phage, and ICE-phage tandem, respectively. Simultaneously, primer pairs (qSCP2/qSCP3, qSCP4/qSCP5, and qSCP2/qSCP5) were designed to amplify the *attP* circular junctions of the respective excised modules. qPCR reactions (20 μl) contained 20 ng of genomic DNA, 0.4 μM each primer, and 1X ChamQ SYBR qPCR Master Mix (Vazyme). The chromosomal single-copy gene *parC* was used as an internal reference to normalize the amount of template DNA. Relative excision and circularization levels were calculated using the 2^−ΔΔCt^ method, representing the fold change in excised or circularized intermediates relative to the WT control under basal conditions.

For oxidative stress gene expression, RNA was extracted from H_2_O_2_-treated cells, reverse transcribed, and analyzed using *parC* as an internal control. The 2^−ΔΔCt^ method was used for relative quantification. Data were shown as means ± SEM. The DNA templates were amplified using a LightCycler96 Real-Time PCR Detection System (Roche) under the following conditions: one cycle of 95°C for 3 min and 40 cycles of 95°C (10 s) and 60°C (30 s). At the end of the run, melt curves were generated to confirm homogeneous products by exposing samples to a final temperature gradient of 65° to 95°C. All assays were performed in triplicate with nuclease-free water as a negative control.

### Expression and purification of Int_ICE_, IntI_Phage_, and IntE_Phage_

The genes encoding IntI_Phage_, IntE_Phage_, and Int_ICE_ were amplified using primers listed in table S1. Plasmids pColdII-*int*_ICE_, pColdII-*intI*_Phage_, and pET32a-*intE*_Phage_ were constructed by restriction digestion with Bam HI and Sal I, or by T4 ligation, to express N-terminal His-tagged fusion proteins. These constructs were transformed into *E. coli* BL21(DE3) for overexpression. Cultures were grown in LB medium supplemented with ampicillin (100 μg/ml) at 37°C to an OD_600_ of 0.4 to 0.6, cooled to 15°C, and induced with 0.6 mM isopropyl-β-d-thiogalactopyranoside at 16°C overnight with shaking at 180 rpm. Cells were harvested by centrifugation at 6000*g* for 15 min at 4°C, resuspended in phosphate-buffered saline (PBS) (pH 7.4), and lysed by sonication on ice. The supernatant was collected after centrifugation at 12,000*g* for 30 min at 4°C, filtered through a 0.45-μm membrane, and subjected to affinity purification using Ni-NTA resin under native conditions. Purified proteins were desalted (PD-10 column, Cytiva), concentrated, and quantified using the Bradford assay before storage at −80°C.

### Electrophoretic mobility shift assay

DNA probes containing *att* sites were PCR amplified (primers in table S1) and incubated with purified integrase proteins in 10 μl of binding buffer [100 mM Hepes (pH 7.6), 5 mM EDTA, 50 mM (NH_4_)_2_SO_4_, 5 mM dithiothreitol, 1% (w/v) Tween 20, and 150 mM KCl] for 30 min at room temperature at protein:DNA molar ratios of 1:5 and 1:10. Reaction mixtures were resolved on 6% nondenaturing polyacrylamide gels in 0.5× tris-borate EDTA (TBE) buffer at 200 V for 45 min. Gels were stained with SYBR Gold in 1× TBE for 20 min, destained in water, and visualized under UV light.

### Filter mating experiments

Conjugative transfer was assessed as previously described (*28*) with modifications to distinguish conjugation from transduction and transformation. Donor and recipient strains were mixed at a 1:10 ratio, concentrated on nitrocellulose membranes placed on THB agar plates, and incubated at 37°C for 8 hours. To rule out phage-mediated transduction, cell-free culture supernatants from donor strains were filtered (0.22-μm pore size) and mixed with recipient cells under identical conditions; no transconjugants were obtained. To exclude natural transformation, DNase I (1 μg/ml) was added to the mating mixture. Cells were resuspended in THB, and transconjugants were selected on agar containing appropriate antibiotics. Three biological and technical replicates were performed. Putative transconjugants were confirmed by PCR (primers in table S1), PFGE, Southern blotting, and WGS.

### HGT via transformation

Donor strains (*S. suis* HCJ3 and SS389; table S2) and recipient strains (*S. suis* P1/7RF and P1/7RFΔrecA) were cultured overnight, diluted to OD_600_ ≈ 0.2, mixed at a 1:10 ratio, washed, and treated with DNase I (1 μg/ml) or PBS for 1 hour at 37°C. Cells were concentrated on nitrocellulose filters on THB agar, incubated for 8 hours at 37°C, and plated on selective media. Three biological and technical replicates were performed for each condition.

### PFGE and Southern blotting

PFGE was performed as previously described (*68*, *69*). Genomic DNA from donors, recipients, and transconjugants was digested with Sma I and separated by PFGE using a CHEF-DR II system (Bio-Rad) in 0.5× TBE at 14°C and 6 V/cm for 20 hours, with pulse times from 1.2 s to 30 s. *S. enterica* serovar Braenderup H9812 digested with Xba I served as a molecular weight standard. For Southern blotting, DIG-labeled probes targeting antimicrobial-resistance genes (ARGs) (primers in table S1) were hybridized and detected using the DIG High Prime DNA Labeling and Detection Starter Kit I (Roche). Images were acquired with a Bio-Rad Gel Doc XR+ system.

### Competition assays

The fitness difference between transconjugants and recipients was investigated by competition assays as described previously (*70*, *71*). Relative fitness was determined by coculturing recipient and transconjugant strains at a 1:1 ratio in THB at 37°C with shaking for 24 hours. Cultures were plated on nonselective and selective media at 0 and 24 hours, and the competitive index (*W*) was calculated as described. Assays included 10 biological replicates.

### Stress susceptibility assays

For oxidative stress, strains were cultured to exponential phase, embedded in soft agar (6 mg/mL agar in THB), and challenged with 2.5 μL of 1 M H_2_O_2_ on a filter disk. Inhibition zones were measured after 48 h at 37°C. For UV sensitivity, cells were exposed to UV light (254 nm, 30 s at 30 cm), serially diluted, and plated for survival quantification. For MMC sensitivity, cultures were treated with 0.5 μg/mL MMC for 30 min, washed, and plated for colony counts.

### Intracellular H_2_O_2_ measurement

Cells treated with 25 mM H_2_O_2_ for 25 min were washed with PBS, and intracellular H_2_O_2_ levels were quantified using the Pierce Quantitative Peroxide Assay Kit (Thermo Fisher Scientific). Three biological replicates were analyzed, and statistical significance was assessed using an unpaired three-tailed t-test.

### RNA-seq and transcriptomic analysis

*S. suis* P1/7RF, cSS389_Phage2, and cAKJ18_Phage were treated with 25 mM H_2_O_2_ for 25 min at exponential phase. RNA was extracted, and libraries were prepared and sequenced on an Illumina HiSeq 2500 platform. Reads were aligned to the reference genome using Bowtie2 (*72*), and gene expression was quantified as FPKM. Differential expression analysis was performed with DESeq2 (*73*) using thresholds of |log_2_(FC)| ≥ 1 and adjusted *P* value < 0.05.

### Genome-wide identification of phage-ICEs

A total of 314,818 bacterial and archaeal genomes from the NCBI RefSeq (accessed December 2023) were analyzed. Viral sequences were identified with VirSorter2 (*74*) (v2.2.4; --min-score 0.5, --min-length 1500), and CheckV (*75*) was used to assess completeness (≥50% complete retained) (fig. S10). Prophages were annotated with geNomad (*76*). ICE/IME elements were predicted using ICEfinder 2.0 (*77*). To identify potential tandem arrangements, we screened for cases where a predicted prophage and an ICE/IME were colocalized on the same contig with an interelement distance of ≤0 bp (indicating direct adjacency or overlapping attachment sites).

The initial 11,012 putative phage-ICE candidates underwent rigorous manual curation to ensure modular integrity. Elements with severe gene decay or lacking core functional modules were excluded. Furthermore, genomic boundaries and attachment (*att*) sites were precisely defined by performing comparative genomic alignments between the tandem-carrying genomes and tandem-free “empty” genomes within the same genus. This integrated pipeline yielded 590 high-confidence phage-ICE tandems (data S2), which were combined with our initial *Streptococcus* dataset for final analysis.

### Sequence similarity network of phage-ICEs

To assess the evolutionary and functional relationships among phage-ICEs, we constructed a sequence similarity network based on protein homology. All-versus-all protein sequence comparisons were performed using MMseqs2 with stringent criteria: *e* value < 10^−4^, sequence identity ≥ 35%, and query coverage ≥ 50%. For each pair of phage-ICEs, best bidirectional hits (BBHs) were identified and used to compute the wGRR (*19*)

$$\text{wGRR}\left(A,\;B\right)=\frac{\sum_{I}^{p}\text{id}\left(A_{i},B_{i}\right)}{\text{min}\left(A,\;B\right)}$$

where *A_i_* and *B_i_* are the *i*th BBH pair of *P* total pairs, the number of genes from the smaller phage-ICE sequence is min(*A*, *B*), and the identity between the BBH pair is id(*A_i_*, *B_i_*). The wGRR varies between 0 (no BBH) and 1 (all genes in an element have an identical BBH in the other). The wGRR scores were used to compute a sequence similarity network of the putative phage-ICE sequence. Genome pairs with wGRR ≤ 0.1 were discarded to reduce the signal’s noise. The construction and clustering of the phage-ICE network were finished in Python; in detail, we used “igraph” to construct the network and “leiden” algorithm in the “leidenalg” package to detect the communities in the network.

### Phylogenetic analysis

A 16*S* rRNA phylogenetic tree was built from 19,083 dereplicated bacterial and archaeal genomes (https://github.com/tseemann/barrnap/). Sequences were aligned with MAFFT (params: --anysymbol --auto) (*78*), trimmed with TrimAl (-automated1) (*58*), and an ML tree was inferred with IQ-TREE (params: -nt AUTO -T AUTO) (*59*). The tree was visualized and annotated using ChiPlot (https://chiplot.online/).

### Functional annotation of the phage-ICE cargo

Open reading frames (ORFs) were predicted with Prodigal (*79*). Virulence factors were identified against the Virulence Factor Database (VFDB) (*80*), and ARGs were annotated using MEGARes 3.0 (*81*), both implemented via DIAMOND (BLASTN; identity ≥ 65%, *e* value < 10^−5^) (*82*). CAZymes involved in carbohydrate metabolism were annotated using dbCAN 3.0 (*83*), which integrates HMMER, DIAMOND, and dbCAN-sub. Functional gene density was calculated as the number of genes per 1 kilo–base pair.

### Quantification and statistical analysis

Data are presented as mean ± SD. Statistical analyses were performed using GraphPad Prism (v10.1.2), R (v4.1.2), and ImageJ (v1.8.0). Details for each experiment are provided in the figure legends.

## Supplementary Materials

### The PDF file includes:

Figs. S1 to S10 Legends for tables S1 and S2 Legends for data S1 to S3 References
