# Supplementary material for: Phage-ICE tandems facilitate bacterial adaptation and antimicrobial resistance spread
Source: Sci Adv. 2026 Oct 9;12(41):eaed3416. doi: 10.1126/sciadv.aed3416 (PMC13651849; doi:10.1126/sciadv.aed3416)
Supplement: Supplementary file 1 [file sciadv.aed3416_sm.pdf]

Science Advances
AAAS

Supplementary Materials for

Phage-ICE tandems facilitate bacterial adaptation and antimicrobial resistance spread

Jinhu Huang *et al.*

Corresponding author: Jinhu Huang, jhuang@njau.edu.cn; Jiale Ma, jialema@njau.edu.cn; Liping Wang, wlp71@163.com; Jinxin Liu, jxnliu@njau.edu.cn

Sci. Adv. **12**, eaed3416 (2026)
DOI: 10.1126/sciadv.aed3416

The PDF file includes:

Figs. S1 to S10
Legends for tables S1 and S2
Legends for data S1 to S3
References

Other Supplementary Material for this manuscript includes the following:

Tables S1 and S2
Data S1 to S3

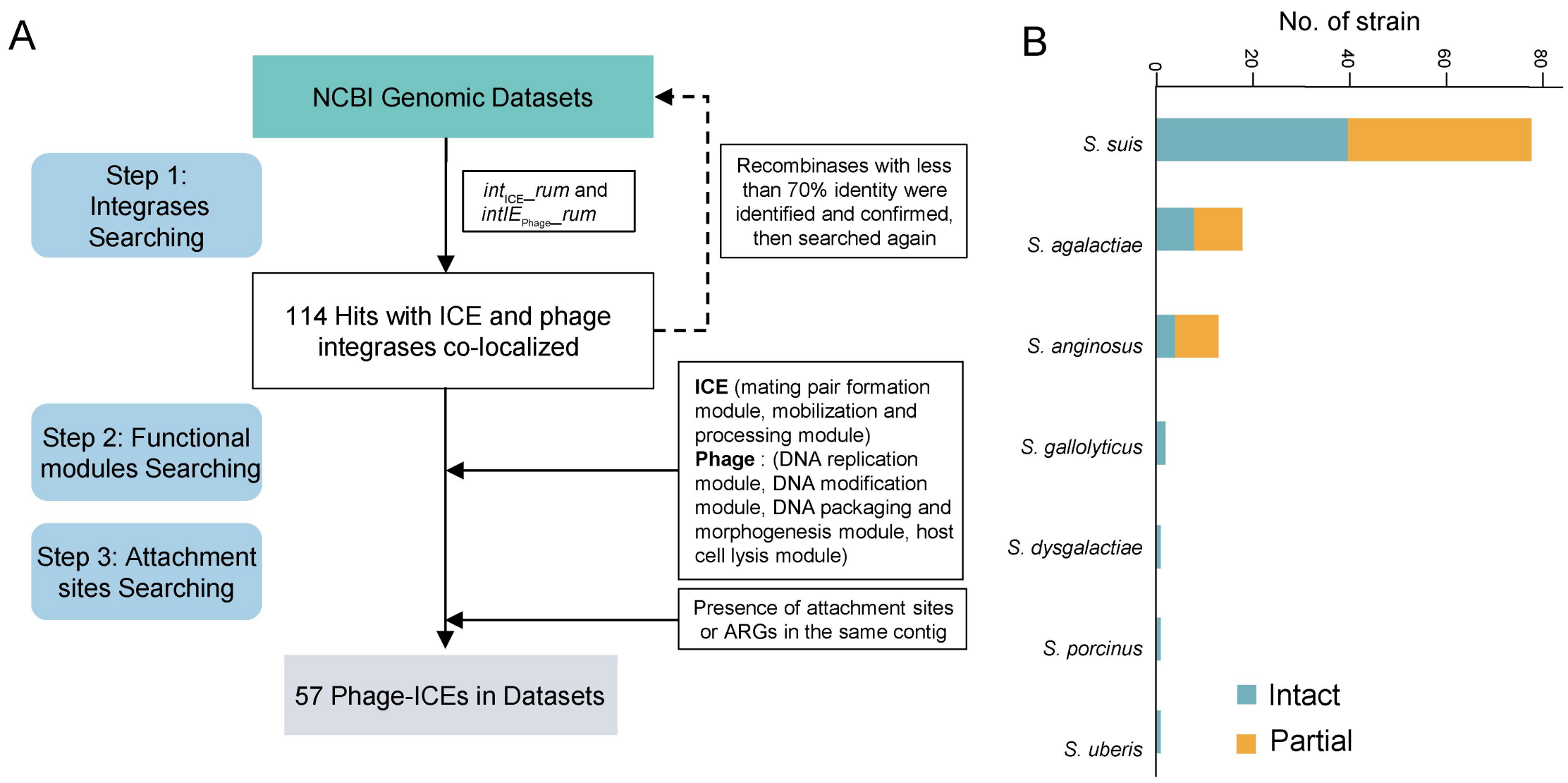

fig. S1. Identification of Phage–ICEs in streptococcal genomes. (A) Schematic of the bioinformatic pipeline used to screen for Phage–ICEs. **(B)** Species distribution of 114 Phage–ICEs identified across seven *Streptococcus* species.

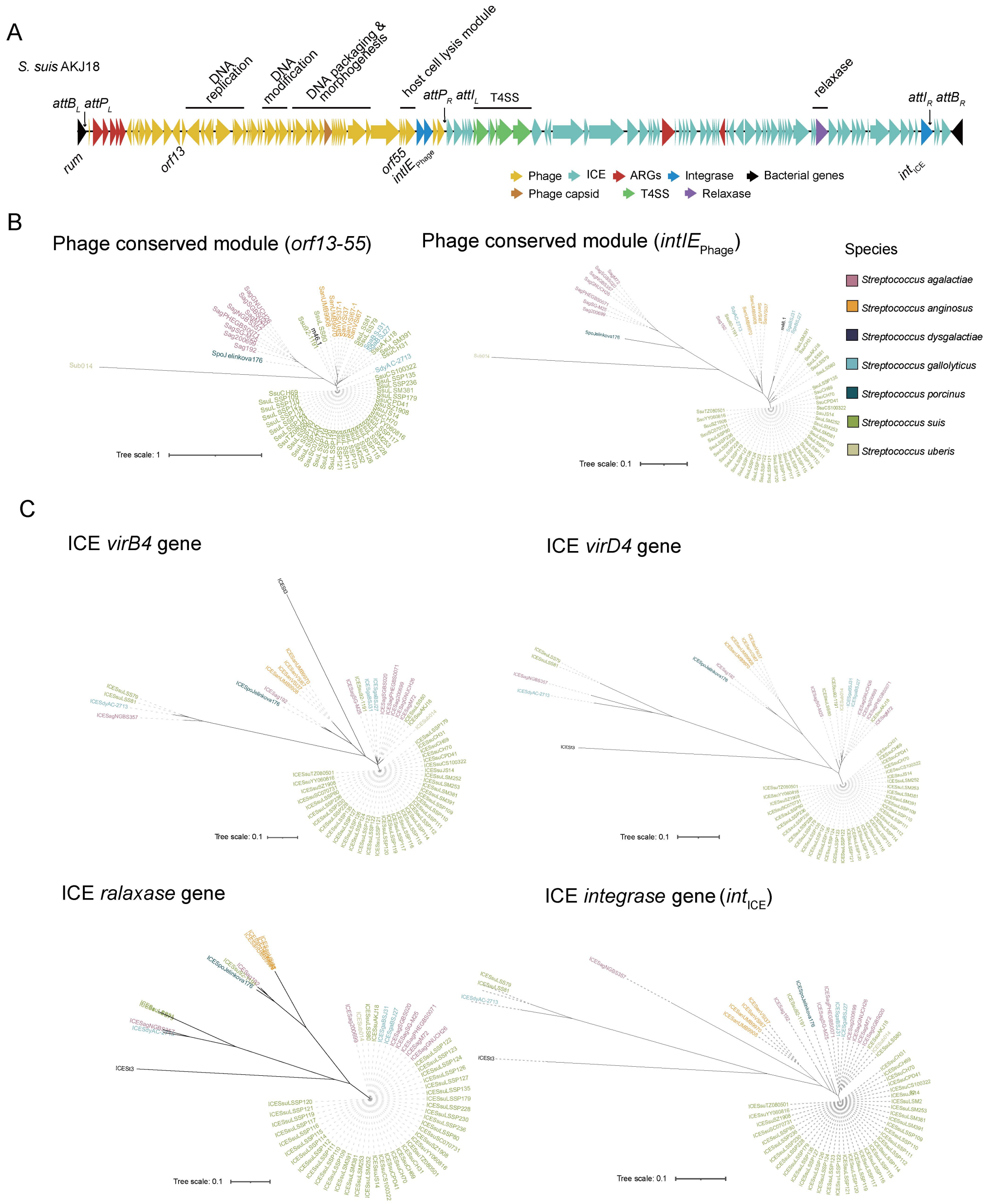

fig. S2. Phylogenetic analysis of phage core genes and ICE backbone genes. (A) Genomic organization of a representative Pha ge–ICE tandem. ICE and phage genes are shown as mint green and yellow arrows, respectively; ARGs, integrases, and chromosomal genes are indicated in red, blue, and black, respectively. **(B)** Phylogenetic tree of phage modules based on nucleotide sequences of five conserved genes (*orf13*–*orf57*; Φm46.1 numbering). Leaves are colored by species. **(C)** Phylogenetic trees of four ICE backbone genes (*virB4*, *virD4*, *relaxase*, and *integrase*). Leaves are colored by species.

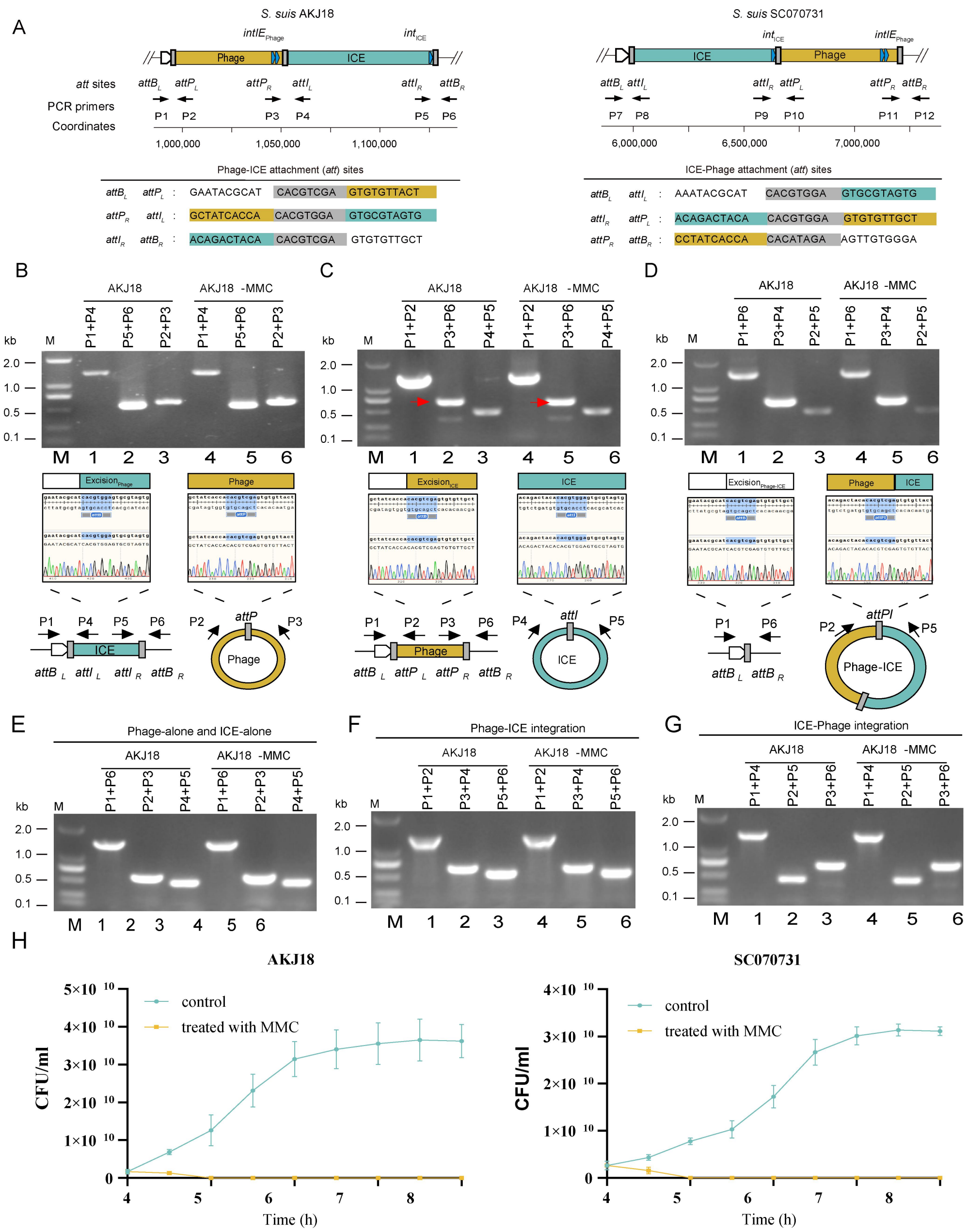

fig. S3. Excision-integration dynamics in *S.suis* AKJ18. (A) Genomic structure of the Phage–ICE region in AKJ18 and SC070731, showing attachment (*att*) sites and primer locations (P1–P12). **(B-G)** PCR detection of integration and excision / circularization junctions resolved on agarose gels. Genomic DNA was extracted from untreated or MMC-treated *S. suis* AKJ18 and used as PCR template. Newly formed junctions were confirmed by Sanger sequencing. **(H)** Viability of *S. suis* AKJ18 and SC070731 treated with or without MMC. Bacterial cultures were adjusted to an OD_{600} of 0.5 at 4 h (defined as time zero), after which they were treated with either 0.5 µg/mL MMC or an equal volume of PBS (control). Samples were collected every 30 minutes for 4 hours to determine colony-forming units (CFU/mL). Data are presented as mean ± SD from three independent experiments.

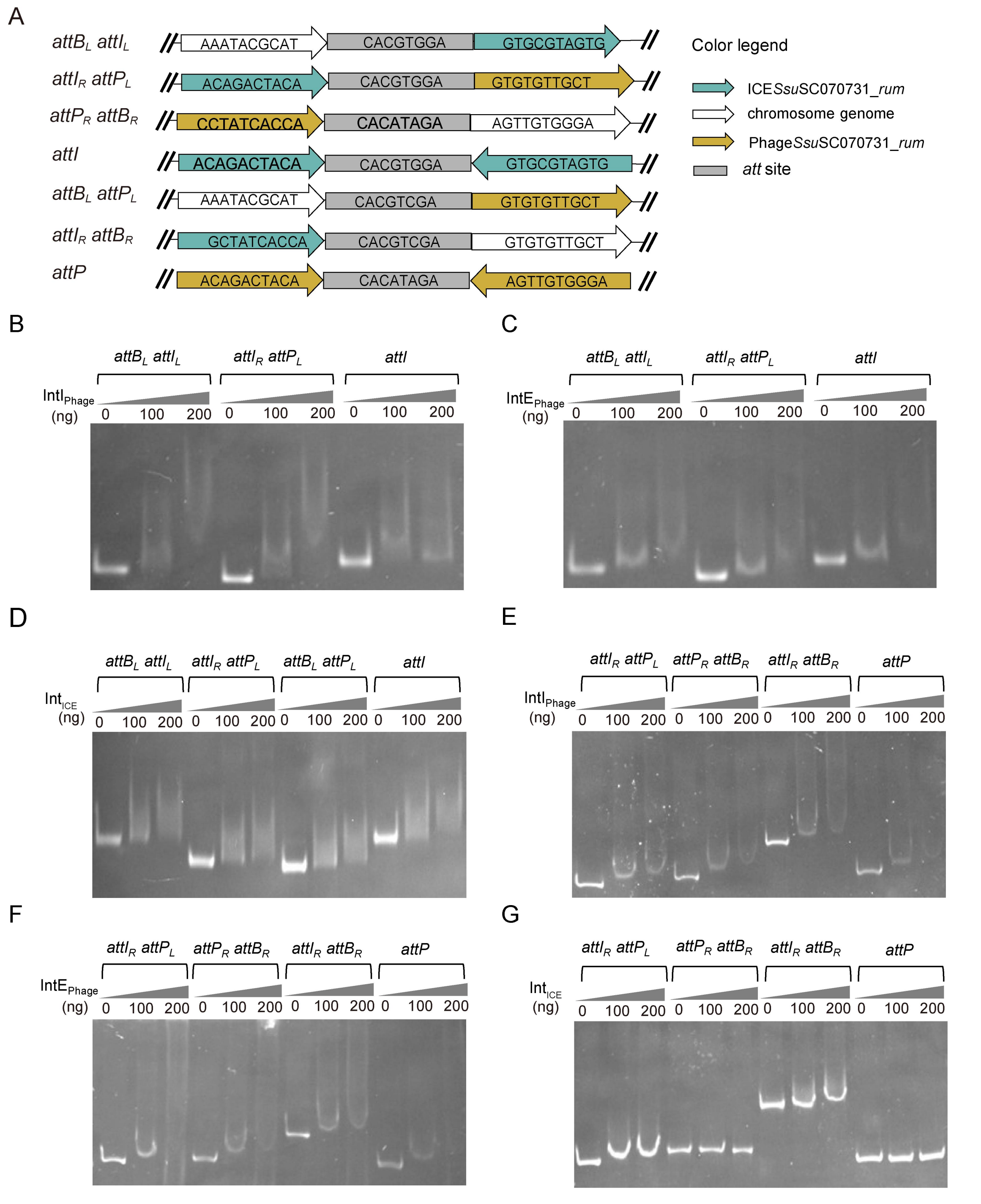

fig. S4. DNA-binding specificity of IntI$_{Phage}$, IntE$_{Phage}$ and Int$_{ICE}$. (A) Representative forms and sequences of *att* sites from *S. suis* SC070731 (ICE–Phage configuration). **(B-G)** EMSA of IntI$_{Phage}$, IntE$_{Phage}$ and Int$_{ICE}$ with indicated *att* sites (Proteins from *S. suis* AKJ18 and *att* site probes from *S. suis* SC070731). IntI$_{Phage}$ and IntE$_{Phage}$ bind all *att* sequences tested, while Int$_{ICE}$ binds its cognate *att* sites but not *attP$_R$ attB$_R$* and *attP*. "(–)": no protein control. (***$P < 0.001$, ****$P < 0.0001$; ns, not significant).

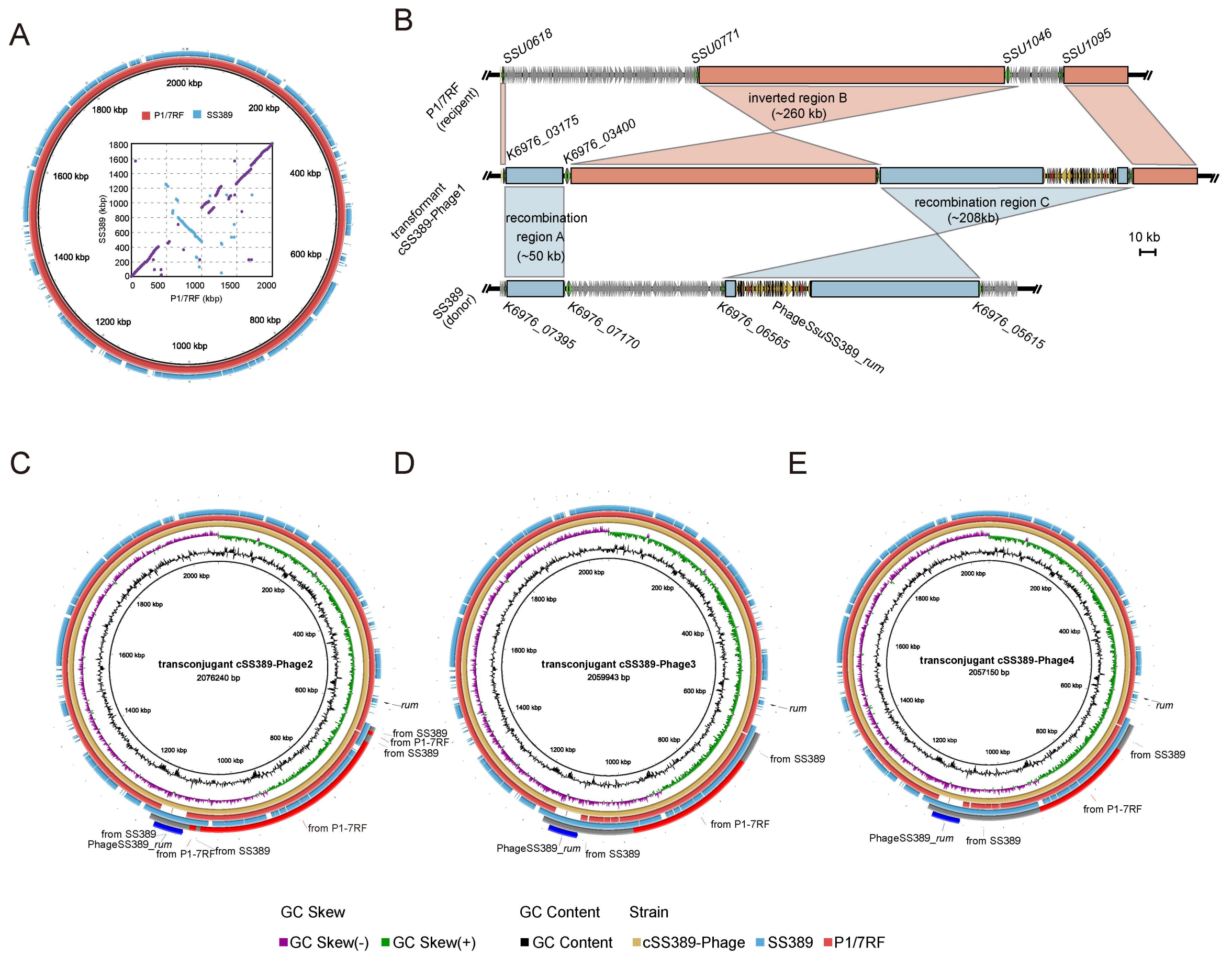

fig. S5. Heterogeneous integration of phage-derived sequences via HR in *S. suis* SS389. (A) Genomic alignment between *S. suis* SS389 (donor, blue) and P1/7RF (recipient, red). The inner box shows MUMmer alignment results. **(B)** Integration structure of transformant cSS389_Phage1. The phage integrated via HR into the *rum* site, with donor-derived regions A (~50 kb) and C (~208 kb) flanking an inverted ~260 kb recipient segment. Color scheme follows Fig. 5, E and F. **(C-E)** Circular genome maps of cSS389_Phage2–4. Each showed distinct HR-derived segments and rearrangements. Inner circle: transformant; middle: recipient P1/7RF; outer: donor SS389. Gray: donor-derived; red: recipient-derived. Black and blue arrows indicate recipient *rum* site and phage insertion, respectively.

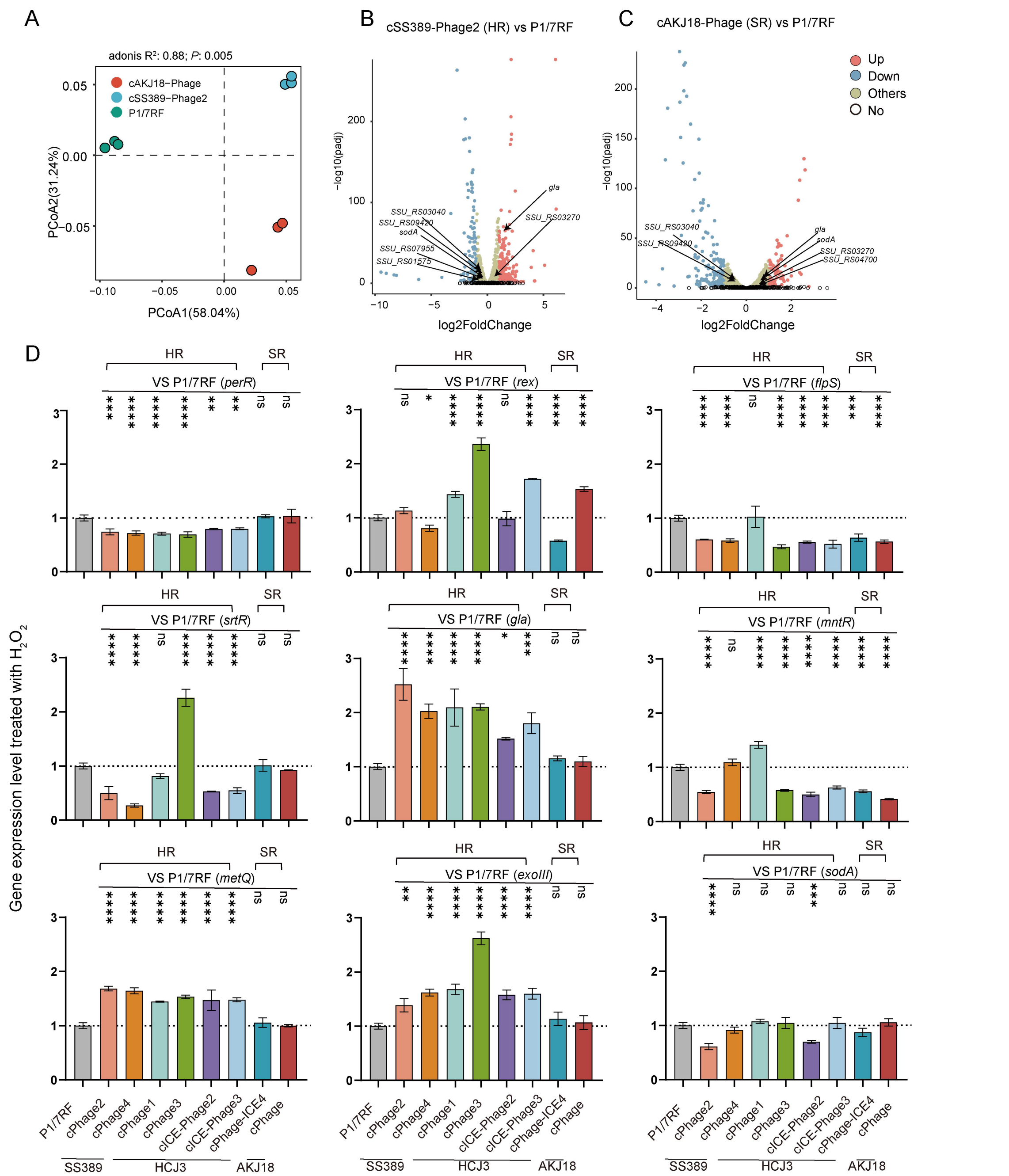

fig. S6. Transcriptional profiling of oxidative stress response in transconjugants. (A) Principal Component Analysis (PCA) of RNA-Seq data from H_2O_2-treated samples. Points represent individual samples: red (cAKJ18_Phage, SR), blue (cSS389_Phage2, HR), green (P1/7RF recipient). **(B-C)** Volcano plots of differentially expressed genes in P1/7RF versus cSS389_Phage2 **(B)** or cAKJ18_Phage **(C)** after H_2O_2 treatment. Red: up-regulated ($log_2FC \geq 1$, padj ≤ 0.05); blue: down-regulated ($log_2FC \leq -1$, padj ≤ 0.05); yellow: non-changing; gray: not significant. **(D)** Expression validation by qPCR of selected oxidative stress genes in HR- and SR-derived transconjugants relative to recipient. Statistical significance: ***P* < 0.01, ****P* < 0.001, *****P* < 0.0001; ns, not significant.

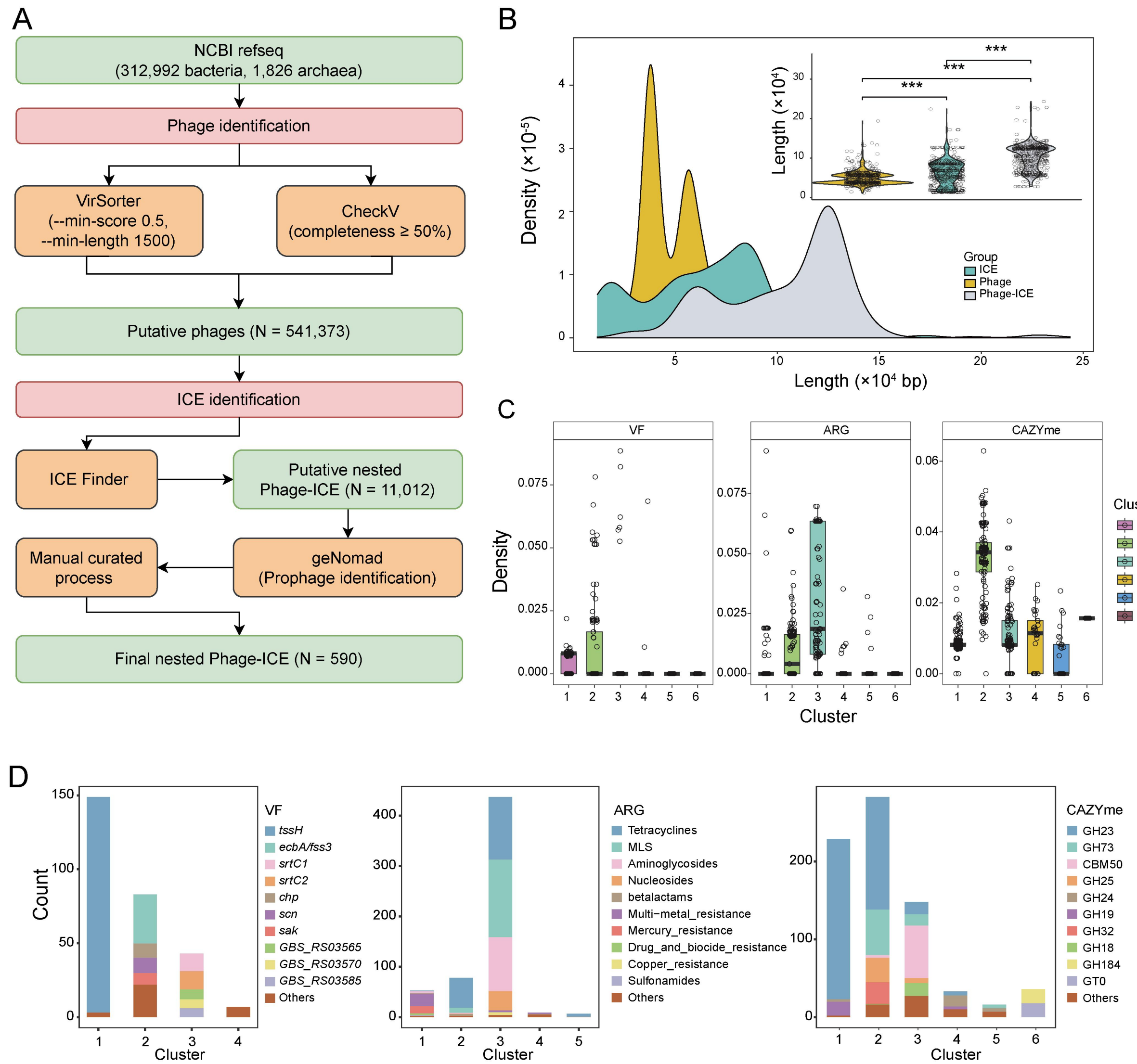

fig. S7. Functional characterization of Phage–ICEs. (A) Bioinformatic workflow for identifying Phage–ICEs in NCBI RefSeq genomes. **(B)** Size distribution of ICE, phage, and Phage–ICE sequences (***$P < 0.001$). **(C)** Density of virulence factors (VFs), antimicrobial resistance genes (ARGs), and CAZyme genes across Phage–ICE communities (top 6 shown). Density is calculated as gene count per 1 kb. **(D)** Distribution of VFs, ARGs, and CAZymes among the top six Phage–ICE communities.

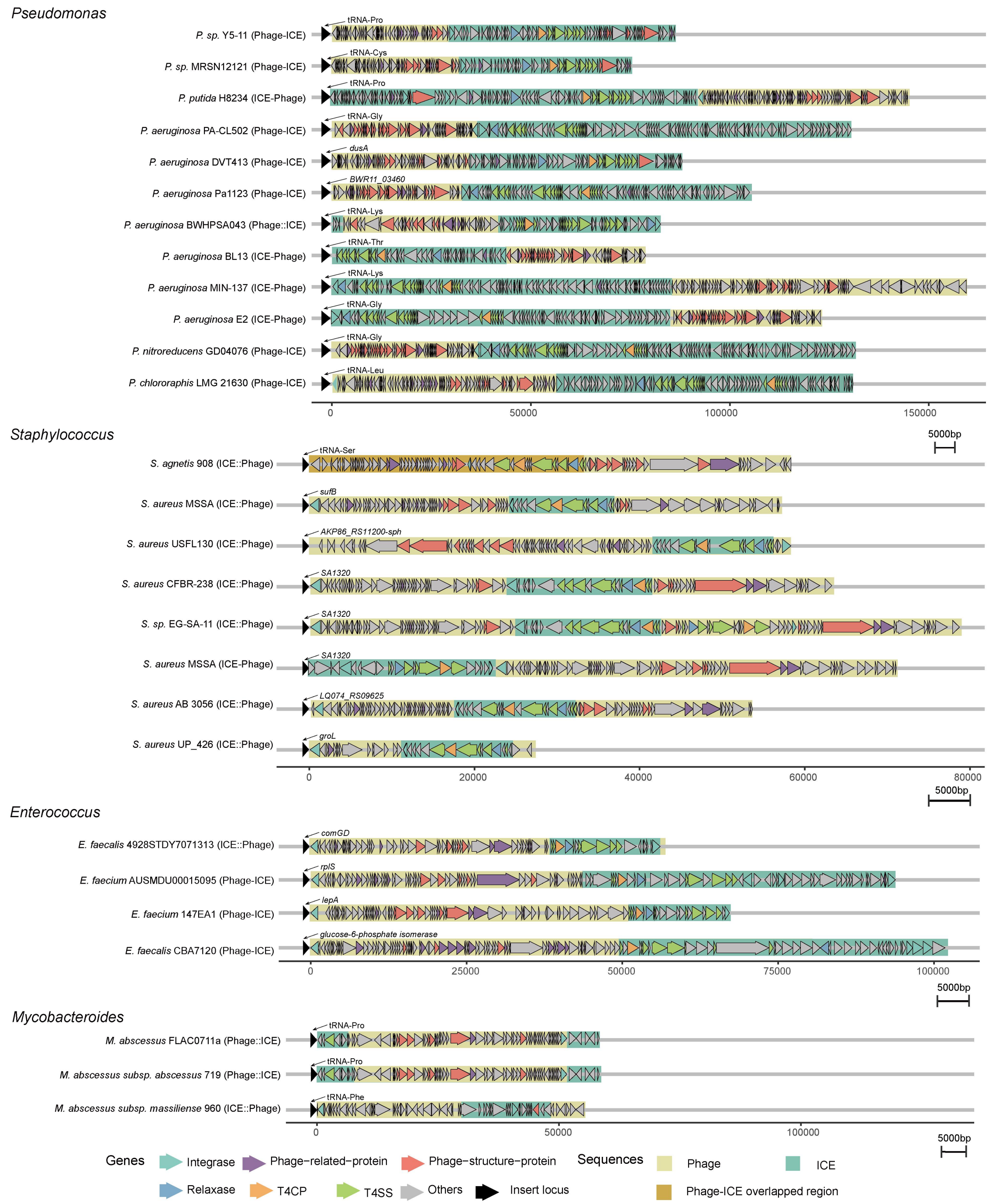

fig. S8. Genetic contexts of Phage–ICEs in four dominant bacterial genera. Genetic maps of representative Phage–ICEs from *Pseudomonas*, *Staphylococcus*, *Enterococcus*, and *Mycobacteroides*. Elements are color-coded: light yellow (phage), teal (ICE), purple (phage-related protein), pink (phage structural protein), golden brown (Phage–ICE overlap), orange (T4CP), green (T4SS), mint blue (integrase), haze blue (relaxase), black (insertion site), gray (other genes).

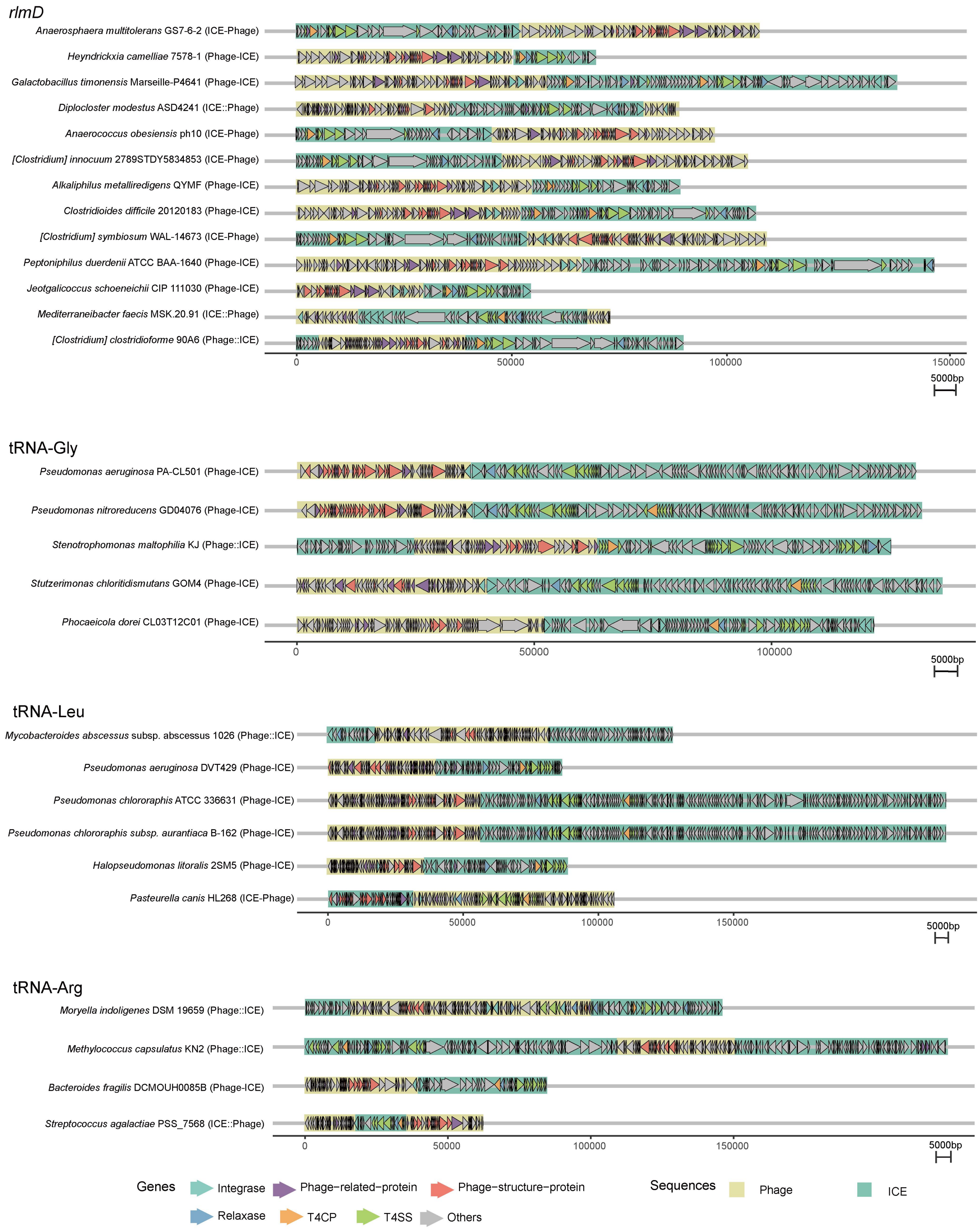

fig. S9. Genomic contexts of Phage–ICEs at the four most frequent integration sites. Genetic maps of Phage–ICEs integrated at *rlmD*, tRNA-Gly, tRNA-Leu, and tRNA-Arg sites. Color scheme follows fig. S8. Representative sequences were selected based on species coverage and integrity.

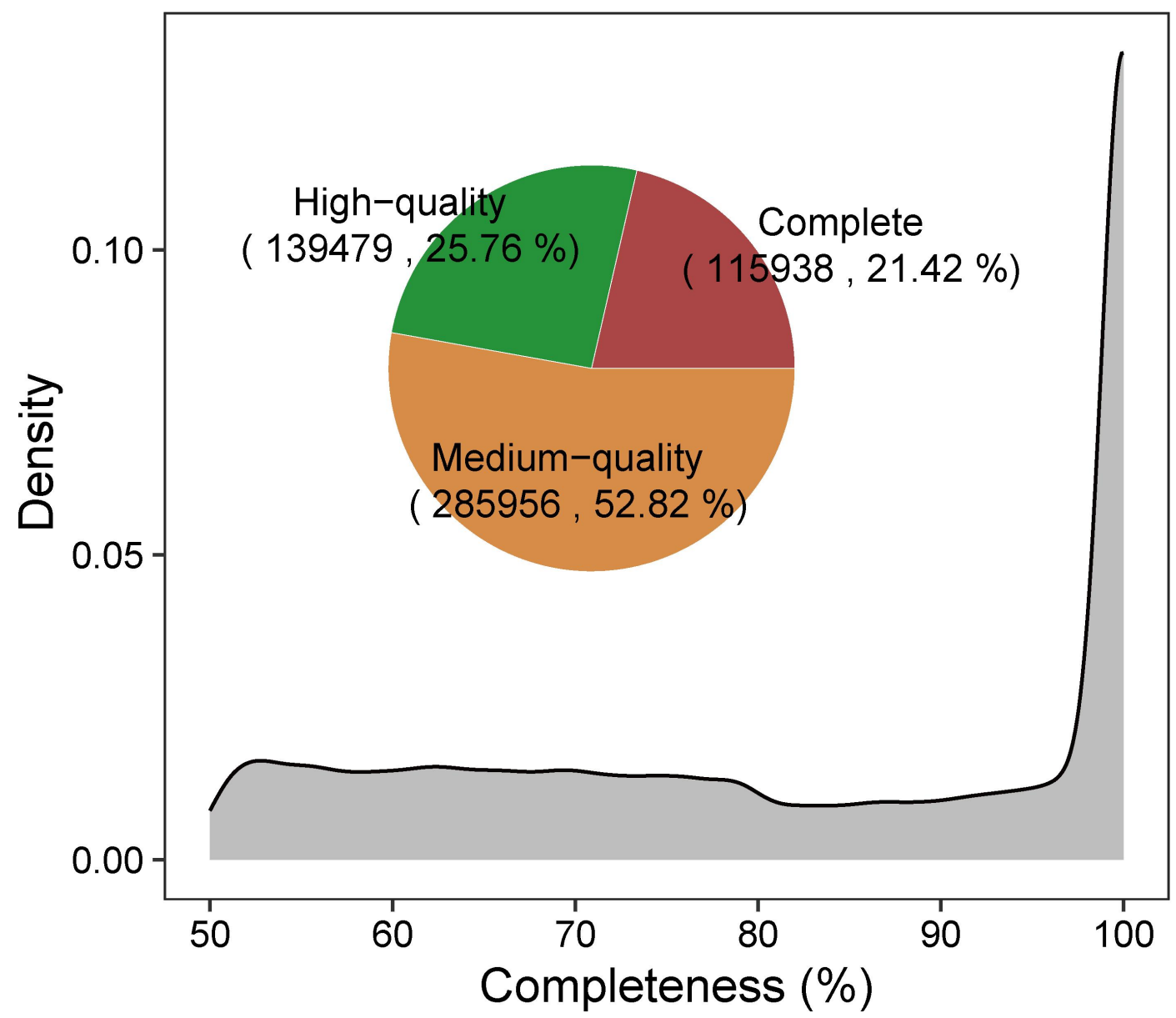

fig. S10. The distribution of CheckV completeness scores for the identified phages. Only sequences with a completeness score above 50% were retained for downstream analysis. Quality categories are defined as: Complete (100% completeness and presence of a direct terminal repeat), High-quality (90% ⩽ completeness ⩽ 100%), Medium-quality (50% ⩽ completeness < 90%).

Table S1. Primers used in this study.

Table S2. Bacterial strains and plasmids used in this study**.**

Data S1. Information of 57 *streptococcal* strains harboring Phage-ICEs with complete boundaries.

Data S2. Information of 612 strains harboring Phage-ICEs.

Data S3. Detailed characterization of insertion loci and flanking sequences for nested Phage::ICE and ICE::Phage configurations.

REFERENCES

1. GBD 2021 Antimicrobial Resistance Collaborators, Global burden of bacterial antimicrobial resistance 1990-2021: A systematic analysis with forecasts to 2050. *Lancet* **404**, 1199–1226 (2024).

2. A. Abbas, A. Barkhouse, D. Hackenberger, G. D. Wright, Antibiotic resistance: A key microbial survival mechanism that threatens public health. *Cell Host Microbe* **32**, 837–851 (2024).

3. R. Maddamsetti, Y. Yao, T. Wang, J. Gao, V. T. Huang, G. S. Hamrick, H.-I. Son, L. You, Duplicated antibiotic resistance genes reveal ongoing selection and horizontal gene transfer in bacteria. *Nat. Commun.* **15**, 1449 (2024).

4. L. S. Frost, R. Leplae, A. O. Summers, A. Toussaint, Mobile genetic elements: The agents of open source evolution. *Nat. Rev. Microbiol.* **3**, 722–732 (2005).

5. J. M. de Sousa, M. Lourenço, I. Gordo, Horizontal gene transfer among host-associated microbes. *Cell Host Microbe* **31**, 513–527 (2023).

6. Y. Che, Y. Yang, X. Xu, K. Břinda, M. F. Polz, W. P. Hanage, T. Zhang, Conjugative plasmids interact with insertion sequences to shape the horizontal transfer of antimicrobial resistance genes. *Proc. Natl. Acad. Sci. U.S.A.* **118**, e2008731118 (2021).

7. M. Ares-Arroyo, C. Coluzzi, J. A. Moura de Sousa, E. P. C. Rocha, Hijackers, hitchhikers, or co-drivers? The mysteries of mobilizable genetic elements. *PLoS Biol.* **22**, e3002796 (2024).

8. S. Castañeda-Barba, E. M. Top, T. Stalder, Plasmids, a molecular cornerstone of antimicrobial resistance in the One Health era. *Nat. Rev. Microbiol.* **22**, 18–32 (2024).

9. T. She, D. Tan, J. L. Balcazar, V.-P. Friman, D. Wang, D. Zhu, M. Ye, M. Sun, S. Yuan, F. Hu, Phage-mediated horizontal transfer of *Salmonella enterica* virulence genes with regulatory feedback from the host. *Imeta* **4**, e70042 (2025).

10. A. S. Lang, A. Buchan, V. Burrus, Interactions and evolutionary relationships among bacterial mobile genetic elements. *Nat. Rev. Microbiol.* **23**, 423–438 (2025).

11. M. K. Yu, E. C. Fogarty, A. M. Eren, Diverse plasmid systems and their ecology across human gut metagenomes revealed by PlasX and MobMess. *Nat. Microbiol.* **9**, 830–847 (2024).

12. R. A. F. Wozniak, M. K. Waldor, Integrative and conjugative elements: Mosaic mobile genetic elements enabling dynamic lateral gene flow. *Nat. Rev. Microbiol.* **8**, 552–563 (2010).

13. J. P. Ramsay, N. Firth, Diverse mobilization strategies facilitate transfer of non-conjugative mobile genetic elements. *Curr. Opin. Microbiol.* **38**, 1–9 (2017).

14. J. Haaber, J. J. Leisner, M. T. Cohn, A. Catalan-Moreno, J. B. Nielsen, H. Westh, J. R. Penadés, H. Ingmer, Bacterial viruses enable their host to acquire antibiotic resistance genes from neighbouring cells. *Nat. Commun.* **7**, 13333 (2016).

15. J. Chen, N. Quiles-Puchalt, Y. N. Chiang, R. Bacigalupe, A. Fillol-Salom, M. S. J. Chee, J. R. Fitzgerald, J. R. Penadés, Genome hypermobility by lateral transduction. *Science* **362**, 207–212 (2018).

16. A. Fillol-Salom, R. Bacigalupe, S. Humphrey, Y. N. Chiang, J. Chen, J. R. Penadés, Lateral transduction is inherent to the life cycle of the archetypical *Salmonella* phage P22. *Nat. Commun.* **12**, 6510 (2021).

17. A. Varble, E. Campisi, C. W. Euler, L. A. Marraffini, Prophage integration into CRISPR loci enables evasion of antiviral immunity in *Streptococcus pyogenes*. *Nat. Microbiol.* **6**, 1516–1525 (2021).

18. L. He, J. B. Patkowski, J. Wang, L. Miguel-Romero, C. H. S. Aylett, A. Fillol-Salom, T. R. D. Costa, J. R. Penadés, Chimeric infective particles expand species boundaries in phage-inducible chromosomal island mobilization. *Cell* **188,** 6636–6653.e17 (2025).

19. E. Pfeifer, J. A. Moura de Sousa, M. Touchon, E. P. C. Rocha, Bacteria have numerous distinctive groups of phage–plasmids with conserved phage and variable plasmid gene repertoires. *Nucleic Acids Res.* **49**, 2655–2673 (2021).

20. E. Pfeifer, E. P. C. Rocha, Phage-plasmids promote recombination and emergence of phages and plasmids. *Nat. Commun.* **15**, 1545 (2024).

21. W. Figueroa, D. Cazares, A. Cazares, Phage-plasmids: Missed links between mobile genetic elements. *Trends Microbiol.* **32**, 622–623 (2024).

22. X. Shan, R. E. Szabo, O. X. Cordero, Mutation-induced infections of phage-plasmids. *Nat. Commun.* **14**, 2049 (2023).

23. S. Nair, C. R. Barker, M. Bird, D. R. Greig, C. Collins, A. Painset, M. Chattaway, D. Pickard, L. Larkin, S. Gharbia, X. Didelot, P. Ribeca, Presence of phage-plasmids in multiple serovars of *Salmonella enterica*. *Microb. Genom.* **10**, 001247 (2024).

24. G. Pavlovic, V. Burrus, B. Gintz, B. Decaris, G. Guédon, Evolution of genomic islands by deletion and tandem accretion by site-specific recombination: ICESt1-related elements from *Streptococcus thermophilus*. *Microbiology* **150**, 759–774 (2004).

25. X. Bellanger, C. Morel, F. Gonot, A. Puymège, B. Decaris, G. Guédon, Site-specific accretion of an integrative conjugative element together with a related genomic island leads to cis mobilization and gene capture. *Mol. Microbiol.* **81**, 912–925 (2011).

26. C. García-Aljaro, E. Ballesté, M. Muniesa, Beyond the canonical strategies of horizontal gene transfer in prokaryotes. *Curr. Opin. Microbiol.* **38**, 95–105 (2017).

27. J. Huang, X. Dai, Z. Wu, X. Hu, J. Sun, Y. Tang, W. Zhang, P. Han, J. Zhao, G. Liu, X. Wang, S. Mao, Y. Wang, D. R. Call, J. Liu, L. Wang, Conjugative transfer of streptococcal prophages harboring antibiotic resistance and virulence genes. *ISME J.* **17**, 1467–1481 (2023).

28. J. Huang, J. Ma, K. Shang, X. Hu, Y. Liang, D. Li, J. Chen, L. Zhang, Z. Chen, X. Wang, J. Zhao, L. Wang, Evolution and diversity of the antimicrobial resistance associated mobilome in *Streptococcus suis*: A probable mobile genetic elements reservoir for other streptococci. *Front. Cell. Infect. Microbiol.* **6,** 118 (2016).

29. M. Tokuda, M. Shintani, Microbial evolution through horizontal gene transfer by mobile genetic elements. *J. Microbial. Biotechnol.* **17,** e14408 (2024).

30. C. M. Johnson, A. D. Grossman, Integrative and conjugative elements (ICEs): What they do and how they work. *Annu. Rev. Genet.* **49**, 577–601 (2015).

31. E. Giovanetti, A. Brenciani, G. Morroni, E. Tiberi, S. Pasquaroli, M. Mingoia, P. E. Varaldo, Transduction of the *Streptococcus* pyogenes bacteriophage φm46.1, carrying resistance genes *mef*(A) and *tet*(O), to other *Streptococcus* species. *Front. Microbiol.* **5**, 746 (2015).

32. C. Smillie, M. P. Garcillán-Barcia, M. V. Francia, E. P. Rocha, F. de la Cruz, Mobility of plasmids. *Microbiol. Mol. Biol. Rev.* **74**, 434–452 (2010).

33. T. Horne, V. T. Orr, J. P. J. Hall, How do interactions between mobile genetic elements affect horizontal gene transfer?. *Curr. Opin. Microbiol.* **73**, 102282 (2023).

34. E. L. Torrance, A. Diop, L. M. Bobay, Homologous recombination shapes the architecture and evolution of bacterial genomes. *Nucleic Acids Res.* **53**, gkae1265 (2024).

35. Y. Wang, T. Dagan, The evolution of antibiotic resistance islands occurs within the framework of plasmid lineages. *Nat. Commun.***15**, 4555 (2024).

36. É. Danchin, A. Charmantier, F. A. Champagne, A. Mesoudi, B. Pujol, S. Blanchet, Beyond DNA: Integrating inclusive inheritance into an extended theory of evolution. *Nat. Rev. Genet.* **12**, 475–486 (2011).

37. L. P. Henry, M. Bruijning, S. K. G. Forsberg, J. F. Ayroles, The microbiome extends host evolutionary potential. *Nat. Commun.* **12**, 5141 (2021).

38. M. Kujawska, D. Seki, L. Chalklen, J. Malsom, R. Kiu, S. Goatcher, I. Christoforou, S. Mitra, L. Crouch, L. J. Hall, Host-specific microbiome and genomic signatures in Bifidobacterium reveal co-evolutionary and functional adaptations across diverse animal hosts. *Cell Host Microbe* **33**, 1502–1517.e13 (2025).

39. D. Berbel, A. González-Díaz, G. L. de Egea, J. Càmara, C. Ardanuy, An overview of macrolide resistance in streptococci: Prevalence, mobile elements and dynamics. *Microorganisms* **10**, 2316 (2022).

40. S. Khedkar, G. Smyshlyaev, I. Letunic, O. M. Maistrenko, L. P. Coelho, A. Orakov, S. K. Forslund, F. Hildebrand, M. Luetge, T. S. B. Schmidt, O. Barabas, P. Bork, Landscape of mobile genetic elements and their antibiotic resistance cargo in prokaryotic genomes. *Nucleic Acids Res.* **50**, 3155–3168 (2022).

41. S. Qin, W. Xiao, C. Zhou, Q. Pu, X. Deng, L. Lan, H. Liang, X. Song, M. Wu, *Pseudomonas aeruginosa*: Pathogenesis,virulence factors,antibiotic resistance,interaction with host,technology advances and emerging therapeutics. *Signal Transduct. Target. Ther.* **7**, 199 (2022).

42. A. Elfadadny, R. F. Ragab, M. AlHarbi, F. Badshah, E. Ibáñez-Arancibia, A. Farag, A. O. Hendawy, P. R. De los Ríos-Escalante, M. Aboubakr, S. A. Zakai, W. M. Nageeb, Antimicrobial resistance of *Pseudomonas aeruginosa*: Navigating clinical impacts, current resistance trends, and innovations in breaking therapies. *Front. Microbiol.* **15**, 1374466 (2024).

43. M. Biggel, P. Moons, M. N. Nguyen, H. Goossens, S. V. Puyvelde, Convergence of virulence and antimicrobial resistance in increasingly prevalent *Escherichia coli* ST131 *papGII*+ sublineages. *Commun. Biol.* **5**, 752 (2022).

44. N. V. Ravin, Replication and maintenance of linear phage-plasmid N15. *Microbiol. Spectr.* **3**, PLAS-0032-2014 (2015).

45. E. Colombi, F. Bertels, G. Doulcier, E. McConnell, T. Pichugina, K. H. Sohn, C. Straub, H. C. McCann, P. B. Rainey, Rapid dissemination of host metabolism anipulating genes via integrative and conjugative elements. *Proc. Natl. Acad. Sci. U.S.A.* **121**, 11 (2024).

46. J. Guglielmini, L. Quintais, M. P. Garcillán-Barcia, F. de la Cruz, E. P. C. Rocha, The repertoire of ICE in prokaryotes underscores the unity, diversity, and ubiquity of conjugation. *PLOS Genet.* **7**, e1002222 (2011).

47. H. Ku, D. Kelk, D. C. Bauer, J. P. S. Sidhu, Phage-plasmid hybrids as vectors for antibiotic resistance in environmental *Escherichia coli*. *Sci. Total Environ.* **959**, 178157 (2025).

48. A. S. Lang, O. Zhaxybayeva, J. T. Beatty, Gene transfer agents: Phage-like elements of genetic exchange. *Nat. Rev. Microbiol.* **10**, 472–482 (2012).

49. J. Botelho, H. Schulenburg, The role of integrative and conjugative elements in antibiotic resistance evolution. *Trends Microbiol.* **29**, 8–18 (2021).

50. S. Sher, H. A. Khan, Z. Khan, M. S. Siddique, D. A. Bukhari, A. Rehman, Bacteriophages: Potential candidates for the dissemination of antibiotic resistance genes in the environment. *Targets* **3**, 25 (2025).

51. V. Libante, M. Dechêne-Tempier, N. Leblond-Bourget, S. Payot, Detection and quantification of conjugative transfer of mobile genetic elements carrying antibiotic resistance genes. *Methods Mol. Biol.* **2815**, 79–91 (2024).

52. R. B. Chanin, P. T. West, J. Wirbel, M. O. Gill, G. Z. M. Green, R. M. Park, N. Enright, A. M. Miklos, A. S. Hickey, E. F. Brooks, K. K. Lum, I. M. Cristea, A. S. Bhatt, Intragenic DNA inversions expand bacterial coding capacity. *Nature* **634**, 234–242 (2024).

53. C. Y. Chin, K. A. Tipton, M. Farokhyfar, E. M. Burd, D. S. Weiss, P. N. Rather, A high-frequency phenotypic switch links bacterial virulence and environmental survival in *Acinetobacter baumannii*. *Nat. Microbiol.* **3**, 563–569 (2018).

54. G. Tonkin-Hill, C. Ruis, S. D. Bentley, K. A. Lythgoe, J. M. Bryant, Within-host bacterial evolution and the emergence of pathogenicity. *Nat. Microbiol.* **10**, 1829–1840 (2025).

55. A. Richter, F. Blei, G. Hu, J. W. Schwitalla, C. N. Lozano-Andrade, J. Xie, S. A. Jarmusch, M. Wibowo, B. Kjeldgaard, S. Surabhi, X. Xu, T. Jautzus, C. B. W. Phippen, O. Tyc, M. Arentshorst, Y. Wang, P. Garbeva, T. O. Larsen, A. F. J. Ram, C. A. M. van den Hondel, G. Maróti, Á. T. Kovács, Enhanced surface colonisation and competition during bacterial adaptation to a fungus. *Nat. Commun.* **15**, 4486 (2024).

56. Z. Wu, W. Wang, M. Tang, J. Shao, C. Dai, W. Zhang, H. Fan, H. Yao, J. Zong, D. Chen, J. Wang, C. Lu, Comparative genomic analysis shows that *Streptococcus suis* meningitis isolate SC070731 contains a unique 105K genomic island. *Gene* **535**, 156–164 (2014).

57. D. M. Emms, S. Kelly, OrthoFinder: Phylogenetic orthology inference for comparative genomics. *Genome Biol.* **20**, 238 (2019).

58. S. Capella-Gutiérrez, J. M. Silla-Martínez, T. Gabaldón, trimAl: A tool for automated alignment trimming in large-scale phylogenetic analyses. *Bioinformatics* **25**, 1972–1973 (2009).

59. L.-T. Nguyen, H. A. Schmidt, A. von Haeseler, B. Q. Minh, IQ-TREE: A fast and effective stochastic algorithm for estimating maximum-likelihood phylogenies. *Mol. Biol. Evol.* **32**, 268–274 (2015).

60. S. Kalyaanamoorthy, B. Q. Minh, T. K. F. Wong, A. von Haeseler, L. S. Jermiin, ModelFinder: Fast model selection for accurate phylogenetic estimates. *Nat. Methods* **14**, 587–589 (2017).

61. K. Tamura, G. Stecher, S. Kumar, MEGA11: Molecular Evolutionary Genetics Analysis Version 11. *Mol. Biol. Evol.* **38**, 3022–3027 (2021).

62. J. Xie, Y. Chen, G. Cai, R. Cai, Z. Hu, H. Wang, Tree Visualization By One Table (tvBOT): A web application for visualizing, modifying and annotating phylogenetic trees. *Nucleic Acids Res.* **51**, W587–W592 (2023).

63. I. Letunic, P. Bork, Interactive Tree Of Life (iTOL): An online tool for phylogenetic tree display and annotation. *Bioinformatics* **23**, 127–128 (2007).

64. M. J. Sullivan, N. K. Petty, S. A. Beatson, Easyfig: A genome comparison visualizer. *Bioinformatics* **27**, 1009–1010 (2011).

65. CLSI, *Performance Standards for Antimicrobial Susceptibility Testing* (CLSI supplement M100, ed. 33, CLSI, 2023).

66. D. Takamatsu, M. Osaki, T. Sekizaki, Thermosensitive suicide vectors for gene replacement in *Streptococcus suis*. *Plasmid* **46**, 140–148 (2001).

67. Z. Wu, C. Wu, J. Shao, Z. Zhu, W. Wang, W. Zhang, M. Tang, N. Pei, H. Fan, J. Li, H. Yao, H. Gu, X. Xu, C. Lu, The *Streptococcus suis* transcriptional landscape reveals adaptation mechanisms in pig blood and cerebrospinal fluid. *RNA* **20**, 882–898 (2014).

68. A. I. Vela, J. Goyache, C. Tarradas, I. Luque, A. Mateos, M. A. Moreno, C. Borge, J. A. Perea, L. Domínguez, J. F. Fernández-Garayzábal, Analysis of genetic diversity of *Streptococcus suis* clinical isolates from pigs in Spain by pulsed-field gel electrophoresis. *J. Clin. Microbiol.* **41**, 2498–2502 (2003).

69. J. Huang, K. Shang, J. Kashif, L. Wang, Genetic diversity of *Streptococcus suis* isolated from three pig farms of China obtained by acquiring antibiotic resistance genes. *J. Sci. Food Agric.* **95**, 1454–1460 (2014).

70. S. Gagneux, C. D. Long, P. M. Small, T. Van, G. K. Schoolnik, B. J. M. Bohannan, The competitive cost of antibiotic resistance in *Mycobacterium tuberculosis*. *Science* **312**, 1944–1946 (2006).

71. O. Kodio, A. C. Georges Togo, Y. D. Sadio Sarro, B. Fane, F. Diallo, A. Somboro, B. Degoga, M. Kone, G. Coulibaly, M. Tolofoudje, S. Bane, M. Sanogo, B. Kone, N. Coulibaly, D. Dabitao, B. Baya, M. Maiga, F. Bougoudogo, F. Samake, S. Dao, S. Doumbia, S. Diallo, B. Diarra, Competitive fitness of *Mycobacterium tuberculosis in vitro*. *Int. J. Mycobacteriol.* **8**, 287–291 (2019).

72. B. Langmead, S. L. Salzberg, Fast gapped-read alignment with Bowtie 2. *Nat. Methods* **9**, 357–359 (2012).

73. M. D. Robinson, D. J. McCarthy, G. K. Smyth, edgeR: A Bioconductor package for differential expression analysis of digital gene expression data. *Bioinformatics* **26**, 139–140 (2010).

74. J. Guo, B. Bolduc, A. A. Zayed, A. Varsani, G. Dominguez-Huerta, T. O. Delmont, A. A. Pratama, M. C. Gazitúa, D. Vik, M. B. Sullivan, S. Roux, VirSorter2: A multi-classifier, expert-guided approach to detect diverse DNA and RNA viruses. *Microbiome* **9**, 37 (2021).

75. S. Nayfach, A. P. Camargo, F. Schulz, E. Eloe-Fadrosh, S. Roux, N. C. Kyrpides, CheckV assesses the quality and completeness of metagenome-assembled viral genomes. *Nat. Biotechnol.* **39**, 578–585 (2021).

76. A. P. Camargo, S. Roux, F. Schulz, M. Babinski, Y. Xu, B. Hu, P. S. G. Chain, S. Nayfach, N. C. Kyrpides, Identification of mobile genetic elements with geNomad. *Nat. Biotechnol.* **42**, 1303–1312 (2024).

77. M. Liu, X. Li, Y. Xie, D. Bi, J. Sun, J. Li, C. Tai, Z. Deng, H.-Y. Ou, ICEberg 2.0: An updated database of bacterial integrative and conjugative elements. *Nucleic Acids Res.* **47**, D660-D665 (2019).

78. K. Katoh, D. M. Standley, MAFFT multiple sequence alignment software version 7: Improvements in performance and usability. *Mol. Biol. Evol.* **30**, 772–780 (2013).

79. D. Hyatt, G.-L. Chen, P. F. LoCascio, M. L. Land, F. W. Larimer, L. J. Hauser, Prodigal: Prokaryotic gene recognition and translation initiation site identification. *BMC Bioinformatics* **11**, 119 (2010).

80. B. Liu, D. Zheng, S. Zhou, L. Chen, J. Yang, VFDB 2022: A general classification scheme for bacterial virulence factors. *Nucleic Acids Res.* **50**, D912-D917 (2022).

81. N. Bonin, E. Doster, H. Worley, L. J. Pinnell, J. E. Bravo, P. Ferm, S. Marini, M. Prosperi, N. Noyes, P. S. Morley, C. Boucher, MEGARes and AMR++, v3.0: An updated comprehensive database of antimicrobial resistance determinants and an improved software pipeline for classification using high-throughput sequencing. *Nucleic Acids Res.* **51**, D744-D752 (2023).

82. B. Buchfink, K. Reuter, H.-G. Drost, Sensitive protein alignments at tree-of-life scale using DIAMOND. *Nat. Methods* **18**, 366–368 (2021).

83. J. Zheng, Q. Ge, Y. Yan, X. Zhang, L. Huang, Y. Yin, dbCAN3: Automated carbohydrate-active enzyme and substrate annotation. *Nucleic Acids Res.* **51**, W115–W121 (2023).

84. X. Zhu, S. Wang, Y. Du, Z. Liang, H. Yao, X. Chen, Z. Wu, A novel aquaporin Aagp contributes to *Streptococcus suis* H_2O_2 efflux and virulence. *Virulence* **14**, 2249789 (2023).

85. Z. Liu, H. Zheng, M. Gottschalk, X. Bai, R. Lan, S. Ji, H. Liu, J. Xu, Development of multiplex PCR assays for the identification of the 33 serotypes of *Streptococcus suis*. *PLOS ONE* **8**, e72070 (2013).

86. S. Ishida, L. H. T. Tien, R. Osawa, M. Tohya, R. Nomoto, Y. Kawamura, T. Takahashi, N. Kikuchi, K. Kikuchi, T. Sekizaki, Development of an appropriate PCR system for the reclassification of *Streptococcus suis*. *J. Microbiol. Methods* **107**, 66–70 (2014).

87. F. C. Tenover, D. J. LeBlanc, P. Elvrum, Cloning and expression of a tetracycline resistance determinant from *Campylobacter jejuni* in *Escherichia coli*. *Antimicrob. Agents Chemother.* **31**, 1301–1306 (1987).

88. C. Palmieri, G. Magi, M. Mingoia, P. Bagnarelli, S. Ripa, P. E. Varaldo, B. Facinelli. Characterization of a *Streptococcus suis tet* (O/W/32/O)-carrying element transferable to major streptococcal pathogens. *Antimicrob. Agents Chemother.* **56**, 4697–702 (2012).

89. L. Chen, J. Huang, X. Huang, Y. He, J. Sun, X. Dai, X. Wang, M. Shafiq, L. Wang, Horizontal transfer of different *erm*(B)-carrying mobile elements among *Streptococcus suis* strains with different serotypes. *Front. Microbiol.* **12**, 628740 (2021).

90. S. B. Hunter, P. Vauterin, M. A. Lambert-Fair, M. S. Van Duyne, K. Kubota, L. Graves, D. Wrigley, T. Barrett, E. Ribot, Establishment of a universal size standard strain for use with the PulseNet standardized pulsed-field gel electrophoresis protocols: Converting the national databases to the new size standard. *J. Clin. Microbiol.* **43**, 1045–1050 (2005).

91. J. Huang, Y. Liang, D. Guo, K. Shang, L. Ge, J. Kashif, L. Wang, Comparative genomic analysis of the ICE*Sa*2603 family ICEs and spread of *erm*(B)- and *tet*(O)-carrying transferable 89K-subtype ICEs in swine and bovine isolates in China. *Front. Microbiol.* **7**, 55 (2016).
